# Discovering *cis-*Regulatory RNAs in *Shewanella* Genomes by Support Vector Machines

**DOI:** 10.1371/journal.pcbi.1000338

**Published:** 2009-04-03

**Authors:** Xing Xu, Yongmei Ji, Gary D. Stormo

**Affiliations:** Department of Genetics, Washington University School of Medicine, St. Louis, Missouri, United States of America; The Hebrew University, Israel

## Abstract

An increasing number of *cis-*regulatory RNA elements have been found to regulate gene expression post-transcriptionally in various biological processes in bacterial systems. Effective computational tools for large-scale identification of novel regulatory RNAs are strongly desired to facilitate our exploration of gene regulation mechanisms and regulatory networks. We present a new computational program named RSSVM (RNA Sampler+Support Vector Machine), which employs Support Vector Machines (SVMs) for efficient identification of functional RNA motifs from random RNA secondary structures. RSSVM uses a set of distinctive features to represent the common RNA secondary structure and structural alignment predicted by RNA Sampler, a tool for accurate common RNA secondary structure prediction, and is trained with functional RNAs from a variety of bacterial RNA motif/gene families covering a wide range of sequence identities. When tested on a large number of known and random RNA motifs, RSSVM shows a significantly higher sensitivity than other leading RNA identification programs while maintaining the same false positive rate. RSSVM performs particularly well on sets with low sequence identities. The combination of RNA Sampler and RSSVM provides a new, fast, and efficient pipeline for large-scale discovery of regulatory RNA motifs. We applied RSSVM to multiple *Shewanella* genomes and identified putative regulatory RNA motifs in the 5′ untranslated regions (UTRs) in *S. oneidensis*, an important bacterial organism with extraordinary respiratory and metal reducing abilities and great potential for bioremediation and alternative energy generation. From 1002 sets of 5′-UTRs of orthologous operons, we identified 166 putative regulatory RNA motifs, including 17 of the 19 known RNA motifs from Rfam, an additional 21 RNA motifs that are supported by literature evidence, 72 RNA motifs overlapping predicted transcription terminators or attenuators, and other candidate regulatory RNA motifs. Our study provides a list of promising novel regulatory RNA motifs potentially involved in post-transcriptional gene regulation. Combined with the previous *cis-*regulatory DNA motif study in *S. oneidensis*, this genome-wide discovery of *cis-*regulatory RNA motifs may offer more comprehensive views of gene regulation at a different level in this organism. The RSSVM software, predictions, and analysis results on *Shewanella* genomes are available at http://ural.wustl.edu/resources.html#RSSVM.

## Author Summary

RNA is remarkably versatile, acting not only as messengers to transfer genetic information from DNA to protein but also as critical structural components and catalytic enzymes in the cell. More intriguingly, RNA elements in messenger RNAs have been widely found in bacteria to control the expression of their downstream genes. The functions of these RNA elements are intrinsically linked to their secondary structures, which are usually conserved across multiple closely related species during evolution and often shared by genes in the same metabolic pathways. We developed a new computational approach to find putative functional RNA elements by looking for conserved RNA secondary structures that are distinguished from random RNA secondary structures in the orthologous RNA sequences from related species. We applied this approach to multiple *Shewanella* genomes and predicted putative regulatory RNA elements in *Shewanella oneidensis*, a bacterium that has extraordinary respiratory and metal reducing abilities and great potential for bioremediation and alternative energy generation. Our findings not only recovered many RNA elements that are known or supported by literature evidence but also included exciting novel RNA elements for further exploration.

## Introduction

RNA is remarkably versatile [1],[2], acting not only as messengers to transfer genetic information from DNA to protein, but also as critical structural components [3] and catalytic enzymes [4],[5] in the cell. More intriguingly, non-coding RNAs (ncRNA) have been found to play important regulatory roles. They can mediate gene expression post-transcriptionally in two ways: one is to serve as *trans*-acting antisense RNAs, such as microRNAs, which hybridize with target mRNAs to silence their expression [6],[7]; the other is to form structural *cis-*elements in the mRNAs, such as riboswitches, which regulate gene expression by mediating transcription termination or translation initiation [8],[9]. The regulatory roles of ncRNAs make them promising drug targets [10] and efficient tools for drug development and gene therapy [11],[12].

In the past a few years, many *cis-*regulatory RNA structural motifs have been identified in prokaryotes [13]–[15]. They are often located in the 5′ untranslated regions (UTR) of the mRNAs and can sense or interact with cognate factors, including proteins, RNAs, small metabolites, or even temperature changes, to mediate transcription attenuation [8], translation initiation [9], or mRNA stability [16]. The functions of the regulatory RNAs are intrinsically tied to their secondary structures, mostly recognizable as stem-loops or pseudoknots. Moreover, regulatory RNAs are often conserved during evolution: similar regulatory RNA elements can be shared by multiple co-regulated genes in the same metabolic pathway, or conserved in orthologous genes across closely related species [17].

Experimental screenings [18] for *cis-*regulatory RNAs are highly labor and time consuming. As demonstrated by previous studies [19],[20], a parallel way is to find good candidates computationally followed by targeted experimental validation. Because functional regulatory RNAs are often evolutionarily conserved in their secondary structures, we can identify them by finding significantly conserved RNA secondary structures in orthologous genes across closely related species. To accomplish this, we need two tools: one is to accurately predict common RNA secondary structures in multiple related sequences, and the other is to distinguish functional RNA secondary structures from random foldings of RNA sequences.

A number of algorithms have been developed for common RNA secondary structure prediction, such as RNAalifold [21], Dynalign [22], comRNA [23], CMFinder [24] and FoldAlign [25],[26]. We recently published a new algorithm, called RNA Sampler [27], for predicting common RNA secondary structures and structural alignments in multiple sequences. Both our study [27] and independent studies from other researchers [28],[29] have demonstrated that RNA Sampler provides more accurate structure predictions and generates better structural alignments on sequences of a wide range of identities than other leading software for similar purposes. Moreover, RNA Sampler runs fast and is feasible for common RNA secondary structure prediction on the genome scale.

Studies have shown that for a single sequence RNA secondary structure alone is not sufficient to distinguish functional RNA from random sequence [30],[31]. However, with the availability of multiple RNA sequences from related species, comparative genomics approaches provide additional power to identify functional RNA structures. One strategy is to design a scoring function for the predicted RNA secondary structures and examine the difference between the score distributions of real structures and randomly permutated structures, as employed by the RNA identification pipeline based on CMfinder [32] or comRNA [23]. But one limitation of such an approach is that the user needs to generate a large number of random sequence sets for each set of real sequences and doing structure predictions on these permutated sequence sets is usually time consuming. Besides, it can be difficult to find a score cutoff to make the call between functional and random RNAs. Another strategy is to train a classification model based on features that can distinguish common structures of known functional RNAs from those of random RNAs and then apply the classification model on the newly predicted common RNA structures to determine whether they are of functional or random RNAs. RNA classification algorithms employing this strategy include QRNA, RNAz and Dynalign+LIBSVM. QRNA [33] classifies a pairwise sequence alignment by the posterior probabilities of three probabilistic models, “RNA”, “Coding” and “Null” (position independent). RNAz [34] and Dynalign+LIBSVM [35] both employ support vector machines (SVM) to build the classification models. To train a classification model, the developer still needs to generate a large number of random sequence sets as the negative training sets and make structure predictions on them, but once the classification model is trained, the user can directly utilize the model to identify functional RNAs without the need to generate, and perform folding of, random sequences. The type of sequences used to train the classification models is essential to their classification performance on new sequences. QRNA and Dynalign+LIBSVM only use tRNAs and rRNAs in their training on RNA structures, and RNAz is trained on multiple RNA gene/motif families from the Rfam database but only uses sequence sets with high identities. To avoid overfitting the classification model to specific classes of RNAs, using training sets that cover a wide range of sequence identities and a variety of RNA families is more desirable. In addition, training the classification model using more accurately predicted RNA common structures and alignments is advantageous for more sensitive classification of functional RNAs from random ones. RNAz uses RNAalifold [36] for common RNA structure prediction. When using sequence alignments as its input, RNAalifold performs poorly in predicting RNA structures on sequence sets of low identities [27]. The structure prediction accuracy of RNAalifold may be improved by using structural alignments, but RNAz might need to be re-trained to use structural alignments.

In this paper, we present a new SVM based functional RNA identifier named RSSVM (RNA Sampler+Support Vector Machine). RSSVM applies a set of features to represent common RNA secondary structures and structural alignments generated by RNA Sampler, which predicts RNA structures more accurately than other approaches [27]–[29]. RSSVM is trained with RNA sets with a wide range of sequence identities from all bacterial RNA motif/gene families in the Rfam database [37]. RSSVM is more sensitive in identifying real functional RNAs than other leading RNA classification programs, including RNAz, Dynalign+LIBSVM and QRNA, at the same false positive rate. We applied RSSVM on multiple *Shewanella* genomes to identify putative *cis-*regulatory RNA motifs in the 5′-UTRs of orthologous genes.

*Shewanella oneidensis* is a facultative, gram-negative γ-proteobacterium. It has extraordinary abilities to use a wide variety of metals and organic molecules as electron acceptors in respiration [38]–[40], which gives it great potential to be applied in bioremediation of both metal and organic pollutants. The complete genomic sequences of *Shewanella oneidensis* and multiple other *Shewanella* species provide good resources for discovering *cis-*regulatory RNAs using comparative genomics approaches. Combining with the recent predictions of putative DNA *cis-*regulatory motifs in *S. oneidensis* [41], we will have a more complete view of gene regulation in *S. oneidensis* at different regulation levels.

## Results

### Comparison of Performance between RSSVM, RNAz, Dynalign+LIBSVM, and QRNA on Test Sets

We examined the performance of RSSVM in identifying RNA regulatory motifs on 1686 positive and 1686 negative test sequence sets (see Methods) and compared its performance with that of RNAz, Dynalign+LIBSVM and QRNA. Both Dynalign+LIBSVM and QRNA only work on two sequences, thus we examined their performance on all unique pairs of RNA sequences for each test set. The sensitivity and false positive rate (FPR) of the predictions were measured by the fractions of true positive classifications on the positive sets and false positive classifications on the negative sets, respectively. For each prediction, RSSVM, RNAz and Dynalign+SVM are able to report an SVM classification probability (*P*) which measures the confidence of the prediction. The higher the *P*-value, the more confident the prediction. A *P*-value cutoff can be selected to call positive predictions. When a lower *P*-value cutoff is used, although more regulatory RNAs can be identified from the positive sets, more negative test sets may be simultaneously misclassified as regulatory RNAs, leading to a higher false positive rate.

**Figure 1:**
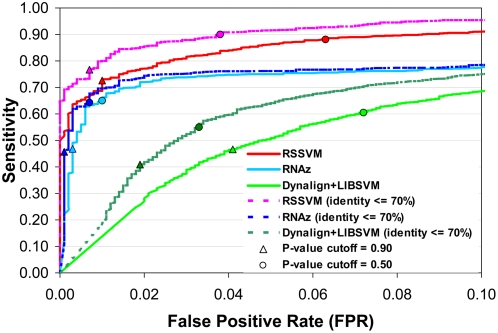
The Receiver Operating Characteristic (ROC) curves of RSSVM, RNAz and Dynalign+LIBSVM on all test sets and on test sets with identities lower than 70%. “▵” and “○” mark the results at *P*-value cutoff of 0.90 and 0.50, respectively. Detailed data for this figure are provided in Dataset S1.

The prediction results from different SVM models at the same *P*-value cutoff are not readily comparable, because their corresponding sensitivities and false positive rates can be significantly different (Figure 1). Thus, to make fair comparisons, we always compare the performance of two programs at the same false positive rate which may be achieved by using different *P*-value cutoffs for different programs (Table S1). The Receiver Operating Characteristic (ROC) curves in Figure 1 demonstrate the prediction sensitivities of RSSVM, RNAz and Dynalign+LIBSVM at different FPRs. RSSVM and RNAz have similar sensitivities on all test sets when the FPR is lower than 0.01. However, when a higher FPR is allowed, RSSVM becomes more sensitive. At the FPR of 0.05, the sensitivities of RSSVM and RNAz are 0.86 and 0.75, respectively. We also compared the performance of RSSVM and RNAz on test sets whose average pairwise sequence identities are lower than 70%. On these test sets, RNAz only has slight improvement in sensitivity in the low FPR range comparing to its performance on all test sets. The prediction sensitivities of RSSVM, however, are about 10% higher than those on all test sets at the same FPRs. RSSVM is much more sensitive than RNAz at any FPR. At the FPR of 0.01, the sensitivity of RSSVM (0.77) is higher than that of RNAz (0.64) by 20% (Dataset S1). The higher prediction sensitivity than RNAz at the same FPR makes RSSVM an alternative choice for the whole genome RNA motif search, as it can find more targets while maintaining a low FPR.

At any FPR, Dynalign+LIBSVM has significantly lower sensitivities than RSSVM and RNAz on all test sets and on test sets with low identities, especially in the range of low FPRs (FPR<0.05) (Figure 1). At the FPR of 0.02, the sensitivities of Dynalign+LIBSVM are only 0.28 and 0.42 on all test sets and on test sets with low identities (<70%), respectively. The mediocre performance of Dynalign+LIBSVM in our tests may be attributed to the following reasons: 1) Dynalign+LIBSVM only uses information from two sequences, but RSSVM and RNAz take advantage of covariance information from multiple sequences; 2) Dynalign+LIBSVM was trained only on tRNAs and 5S rRNAs, which may cause overfitting of its classification model to these RNA families. In fact, we did observe a much higher classification sensitivity of Dynalign+LIBSVM on test sets comprising tRNAs and 5S rRNAs than on all test sets at the same FPRs (data not shown). For whole genome RNA motif scan, an ideal tool is required to have a high sensitivity and a low false positive rate. Dynalign+LIBSVM might not be a good choice for large scale scan of RNA motifs.

QRNA does not provide a similar measurement of *P*-value for its predictions, thus we are not able to generate its ROC curve. But on all test sets, the overall FPR of QRNA is 0.05. At this FPR, RSSVM has a significantly higher sensitivity (0.86) than QRNA (0.51) (Table S1).

**Figure 2:**
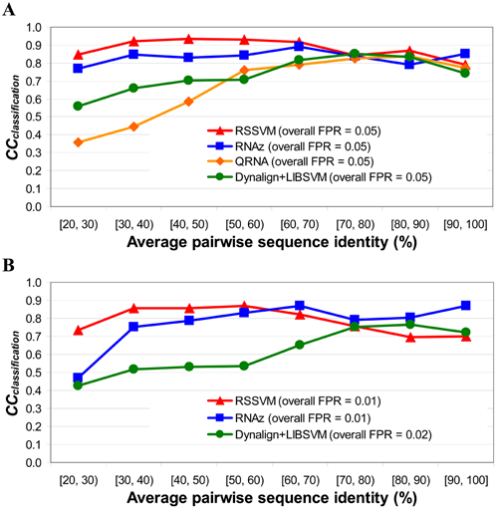
The Correlation Coefficients of RNA classification (*CC_classification_*) by RSSVM, RNAz, Dynalign+LIBSVM and QRNA on test sets with different sequence identities (detailed values are in Table S1). (A) At the overall FPR of 0.05. (B) At the more stringent overall FPR of 0.01 or 0.02. The lowest possible FPR that Dynalign+LIBSVM can achieve is 0.02.

We further evaluated the performance of RSSVM on test sets with different ranges of average sequence identities. We use correlation coefficient (*CC_classification_*), the geometric mean of the classification sensitivity and (1−FPR), to measure the overall performance of RSSVM in each identity range. Because the overall FPR of QRNA on all test sets is 0.05, to make fair comparisons, we use different *P*-value cutoffs for RSSVM, RNAz and Dynalign+LIBSVM to achieve the same FPR of 0.05 on all test sets. As shown in Figure 2A, all algorithms have similar performance on test sets with high identities (≥70%), but RSSVM significantly outperforms all the other algorithms on test sets with low identities (<70%). In general, all tested algorithms tend to have lower FPRs on sequence sets with low identities (<70%) than with high identities (≥70%) (Table S1). The increases in FPRs on high-identity sets may be mainly due to the loss of covariant mutations in the structures. Although Dynalign+LIBSVM and QRNA have low FPRs on low-identity sets, they also make few positive predictions in those sets, leading to low sensitivities.

At the more stringent overall FPR of 0.01 on all test sets, RSSVM (0.68) and RNAz (0.65) have almost the same overall prediction sensitivity (Table S1), and both perform significantly better than Dynalign+LIBSVM, whose lowest possible overall FPR is 0.02 (Figure 2B). However, RSSVM and RNAz outperform each other in different identity ranges. RSSVM is much more sensitive on sequence sets with identities lower than 60%, but RNAz performs better on sequence sets with high identities (≥60%), while both algorithms maintain low FPRs in all identity ranges.

Overall, for the best performance, RNAz, Dynalign+LIBSVM and QRNA are in favor of sequence sets with high identities. RSSVM, however, has consistent and more sensitive performance on the low-identity sets while keeping the same FPRs. These programs can complement each other for the best performance in identifying regulatory RNAs on sequences with a wide range of identities.

**Figure 3:**
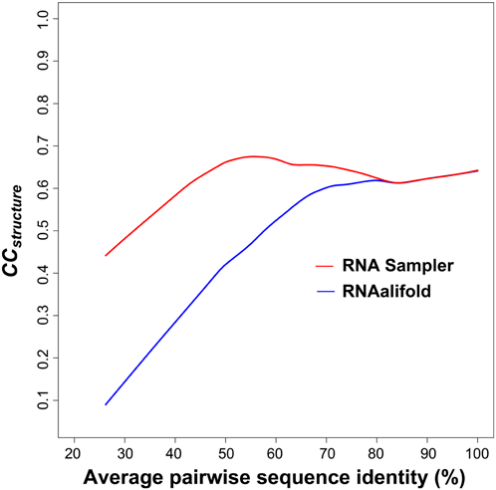
The Correlation Coefficients of predicted structures (*CC_structure_*) by RNA Sampler and RNAalifold, the corresponding core algorithms used by RSSVM and RNAz, respectively, for predicting common RNA structures, on test sets with different sequence identities.

Three major improvements may contribute to the better performance of RSSVM compared to RNAz in identifying regulatory RNAs, especially on test sets whose identities are lower than 70%. The first improvement is using the more accurately predicted common RNA secondary structures by RNA Sampler. The accuracy of predicted structures can be measured by the correlation coefficient of structure prediction (*CC_structure_*), which approximates the geometric mean of the sensitivity and specificity of predicted base pairings [27]. RNA Sampler and RNAalifold are the corresponding core algorithms used by RSSVM and RNAz for predicting common RNA secondary structures, respectively. As shown in Figure 3, RNA Sampler gives similar performance to RNAalifold on the high-identity sequence sets (≥80%) but makes much more accurate structure predictions on the low-identity sets (<80%). The more accurately predicted structures and better alignments by RNA Sampler provide a better start point for RSSVM to identify RNA motifs. Second, the additional features used by RSSVM (see Methods), such as the *SCI* scores calculated based on common structures predicted by RNA Sampler, the information content (IC) which grasps the information of sequence conservation, and the mutual information (MI) which represents covariant mutations in the structural alignments, allow it to generate better SVM models to separate regulatory RNA motifs from shuffled ones, especially on sequence sets with low identities (Figure 1). Third, RSSVM is trained on sequence sets of a wider variety of RNA families and a broader range of sequence identities.

In addition, because the common structures predicted by RNA Sampler are more accurate in general, they may provide insightful hints for inferring the functions of the predicted RNA motifs and guiding the design of experimental validation.

### Prediction of Regulatory RNAs in 5′-UTRs of *Shewanella* Genomes

As many known bacterial regulatory RNA sites are located in the 5′-UTR sequences and often conserved during evolution, we applied RSSVM, RNAz and QRNA on multiple *Shewanella* genomes to identify potential regulatory RNA motifs in the 5′-UTR regions. We retrieved 1002 sets of UTR sequences of orthologous genes from five related *Shewanella* genomes. The average pairwise sequence identities of the UTR sets range from 25% to 88%, with a mean of 45% and median 42%. The majority of the sequence sets are in the identity range of 40–70%, which is ideal for RSSVM to identify functional RNA motifs. We examined each set of UTR sequences in three overlapping windows that cover the regions of −250 to −100, −200 to −50, and −150 to 20 (1 corresponds to the translation start site). For each UTR set, we report the classification result from the window with the best SVM probability for RSSVM or RNAz. We chose *P*≥0.95 and *P*≥0.50 as the confidence probability cutoffs for RSSVM and RNAz, respectively, which give the same overall false positive rate of 0.01 on all test sets. For QRNA, we classified a set as regulatory RNA if more than two pairwise alignments of the sequences were identified as “RNA”.

The total numbers of predicted regulatory RNA motifs by different approaches are listed in Table 1. Of the 1002 orthologous UTR sets, RSSVM, RNAz and QRNA predicted 166, 109 and 112 putative regulatory RNA motifs, respectively. The sensitivities of the predictions can be estimated by the fraction of correctly predicted known RNA motifs/genes. By scanning the orthologous UTR sets with all known bacterial RNA motif models from the Rfam database using the RNA motif searching software Infernal [42], we obtained 19 known RNA motifs that gave infernal scores higher than 10 bits and occurred in at least two orthologous sequences of a UTR set. 6 of the 19 RNA motifs have orthologous sequences from *S. oneidensis* and *E. coli* in the Rfam seed alignments. RSSVM, RNAz and QRNA successfully detected 17, 16 and 11 of these 19 known RNA motifs, respectively, and the three approaches combined discovered 18 known RNA motifs (Table 2). It suggests that RSSVM and RNAz have similar sensitivities and both methods are able to discover more known motifs than QRNA. The one missed by all three approaches is the S15 mRNA leader sequence which contains alternative pseudoknot and stem-loop structures. If we slightly lower the *P-*value cutoff for RSSVM to 0.9, it is able to identify the RNA motif in the S15 UTR set. The success of identifying almost all known RNA motifs in the studied sequence sets demonstrates the high sensitivity of RSSVM.

**Table 1:** Numbers of predicted regulatory RNAs with supporting evidence by RSSVM, RNAz and QRNA in the 1002 orthologous 5′-UTRs of five *Shewanella* species.

	RSSVM (FPR = 0.01)	RNAz (FPR = 0.01)	QRNA
Total number of predicted regulatory RNAs	166	109	112
False positives on shuffled sequences	0	0	13
Matching known RNA motifs in Rfam a (**19**)^d^	17	16	11
Overlapping with predicted transcription terminators or attenuators	72	49	40
Overlapping with predicted transcription terminators b (**106**)^d^	62	42	31
Overlapping with predicted transcription attenuators c (**123**)^d^	56	37	32
With literature support	21	11	7

a We searched all the orthologous UTRs with Infernal using all bacterial RNA motif models from Rfam, and 19 known RNA motifs gave Infernal scores higher than 10 bits and occurred in at least two orthologous sequences of a UTR set. 6 of the 19 RNA motifs have orthologous sequences from *S. oneidensis* and *E. coli* in the Rfam seed alignments.

b Putative transcription terminators predicted by Rnall [43].

c Putative transcription attenuators predicted by a previous comparative genomics study [44].

d Numbers in the parentheses are the total numbers of known RNA motifs or predicted transcription terminators/attenuators in the 1002 *Shewanella* 5′-UTR sequence sets.

**Table 2:** Predicted regulatory RNAs that match the known *cis-*regulatory RNA elements or genes in the Rfam database.

Rank^a^	GI	RSSVM^b^	RNAz^b^	QRNA^c^	Gene Name	Gene Product	Matching RNA Family in Rfam		
1	**24349136**	**1.000**	**1.000**	+^d^	trpE	anthranilate synthase component I	RF00513	Trp_leader	RNA element
3	**24346870**	**1.000**	**1.000**	+^d^	SO1202	conserved hypothetical protein	RF00005	tRNA	tRNA
4	**24351250**	**1.000**	**0.998**	+^d^	SO4727	conserved hypothetical protein	RF00558	L20_leader	RNA element
6	**24347627**	**1.000**	**0.998**		ppiD	peptidyl-prolyl *cis-*trans isomerase D	RF00506	Thr_leader	RNA element
7	**24349634**	**1.000**	**0.997**	+^d^	thrA	aspartokinase I/homoserine dehydrogenase, threonine-sensitive	RF00506	Thr_leader	RNA element
8	**24347975**	**1.000**	**0.997**	+	hisG	ATP phosphoribosyltransferase	RF00514	His_leader	RNA element
16	**24347418**	**1.000**	**0.523**		rpsB	ribosomal protein S2	RF00127	t44 RNA	RNA gene
34	**24346699**	**1.000**	**1.000**	+	SO1071	conserved hypothetical protein	RF00080	yybP-ykoY	Riboswitch
39	**24347085**	**1.000**	**0.997**	+	pheA	chorismate mutase/prephenate dehydratase	RF00513	Trp_leader	RNA element
64	**24346616**	**1.000**	**1.000**	+	SO1007	conserved hypothetical protein	RF00168	Lysine	Riboswitch
73	**24348868**	**0.999**	0.240	+	Rne	ribonuclease E	RF00370	sroD RNA	RNA gene
93	**24346037**	**0.997**	**0.678**		SO0547	conserved hypothetical protein	RF00522	PreQ1	Riboswitch
100	**24348781**	**0.995**	**0.986**		SO2715	TonB-dependent receptor	RF00059	TPP	Riboswitch
117	**24350326**	**0.989**	**0.765**	+^d^	lysC	aspartokinase III, lysine-sensitive	RF00168	Lysine	Riboswitch
120	**24348446**	**0.989**	0.420		thiC	thiamin biosynthesis protein ThiC	RF00059	TPP	Riboswitch
125	**24347051**	**0.987**	**0.969**		nadB	L-aspartate oxidase	RF00522	PreQ1	Riboswitch
133	**24346318**	**0.982**	**0.690**		SO0774	5-formyltetrahydrofolate cyclo-ligase family protein	RF00013	6S RNA	RNA gene
195	**24346874**	0.903	0.014		rpsO	ribosomal protein S15	RF00114	S15 leader	RNA element
302	**24346370**	0.661	**0.820**	+	SO0815	TonB-dependent receptor C-terminal domain protein	RF00174	Cobalamin	Riboswitch
**Total counts**		**17**	**16**	**11**					

a The rank is based on the *P*-value of RSSVM.

b Bold fonts represent predictions above the *P*-value cutoff for RSSVM (0.95) or RNAz (0.50).

c “+” represent QRNA predictions that fit the “RNA” model in at least two pairwise alignments.

d The shuffled sequences were identified as “RNA” by QRNA.

**Figure 4:**
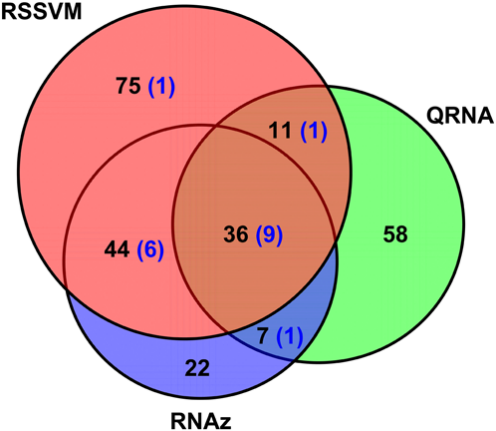
The Venn-diagram of the numbers of predicted regulatory RNAs by RSSVM, RNAz and QRNA. The numbers in the parentheses are of the predictions matching known RNA motifs.

The predictions by the three approaches overlap significantly with each other, as shown in the Venn diagram in Figure 4. 36 RNA motifs are identified by all three approaches, including 9 matching the known RNA motifs. This suggests that consensus predictions by all approaches may have high specificity. RSSVM and RNAz have additional 44 predicted motifs in common, and 6 of them are known motifs. QRNA has additional 11 and 7 motifs overlapping with the predictions by RSSVM and RNAz, respectively, including 2 matching known motifs. These results suggest that predictions cross-validated by different approaches are more likely to be real. Although a large fraction of the predictions by RSSVM and RNAz overlap, 2 and 1 known RNA motifs are identified only by RSSVM and RNAz, respectively, suggesting that combining predictions from different approaches may find more real RNA regulatory motifs. RSSVM made more predictions than RNAz. Besides the 80 predictions in common, 86 and 29 motifs were identified specifically by RSSVM or RNAz, respectively. The overall sequence identity of the commonly predicted sets by RSSVM and RNAz (mean 50%) is significantly higher than that of the predicted sets only by RSSVM or RNAz (mean 41%), with *t-*test *p-*values of 7×10^−7^ between the common and RSSVM specific predictions and 1×10^−3^ between the common and RNAz specific predictions, respectively. As seen in Figure S1, 87% of the sets predicted only by RSSVM or RNAz have sequence identities lower than 50%, while only 60% of the commonly predicted sets have identities lower than 50%. 5% more of the RNAz specific predictions are in the high-identity region (≥60%) than the RSSVM specific predictions. As demonstrated with the test sets, RNAz performs better on sequences of high identities, which is consistent with the observation that majority of the RNAz predictions, especially those in common with the RSSVM predictions, have higher identities than the RSSVM specific predictions. The fact that RSSVM gives more independent predictions than RNAz further demonstrates that RSSVM is more sensitive than RNAz on the low-identity sequence sets.

The specificity, the fraction of correct predictions, is difficult to accurately measure because of the poor knowledge on RNA motifs in *S. oneidensis*. We use the false positive predictions on shuffled sequences to evaluate whether the RNA motifs could be predicted by chance. The RNA Sampler structural alignments or ClustalW alignments of orthologous UTR sets were shuffled using the same approach that generated the negative training and test sets described in Methods and were used as negative controls for RSSVM and RNAz/QRNA, respectively. Both RSSVM and RNAz did not report any RNA motifs in these shuffled sequences, but QRNA had 13 false positive predictions. These results are consistent with the performance of these three approaches on the test sets, with QRNA tending to have more false positives than RSSVM and RNAz.

### Predictions with Supporting Evidence

Besides predictions that match Rfam motifs, we can also assess the accuracy of our predictions by comparing them to other independent types of predictions and to published reports of regulatory motifs or genes undergoing post-transcriptional regulation.

#### Predicted transcription terminators/attenuators

As transcription attenuation is a common regulatory mechanism for RNA motifs in the 5′-UTRs, we checked whether the orthologous UTR sets contain any putative rho-independent transcription terminators predicted by Rnall [43] or putative transcription attenuators predicted by a previous comparative genomics study [44]. Although these predictions are not experimentally verified, agreements between different approaches may provide extra confidence in the predictions. In the 1002 orthologous UTR sets we studied, Rnall predicted 106 putative transcription terminators that are conserved in *S. oneidensis* and at least one other *Shewanella* species. 62 of 166 (37%) and 42 of 109 (39%) RNA motifs predicted by RSSVM and RNAz, respectively, overlap with these predicted transcription terminators. It indicates that the putative transcription terminators are significantly enriched in the predictions by RSSVM and RNAz, corresponding to the hypergeometric p-values of 4.0×10^−26^ and 5.3×10^−17^, respectively. In a previous study, Merino and Yanofsky [44] searched the upstream regions of predicted transcription units for transcription attenuators in 180 bacterial genomes, including *S. oneidensis*. They predicted 449 transcription attenuators in *S. oneidensis*, 123 of which are located in the 1002 orthologous UTR sets that we studied. 56 (34%) and 37 (34%) of the predictions by RSSVM and RNAz match the predicted transcription attenuators, showing significant enrichment with the hypergeometric *p*-values of 2.5×10^−16^ and 1.8×10^−10^, respectively. In both cases, RSSVM's predictions show higher enrichment than RNAz's predictions for putative transcription terminators or attenuators. In total, 72 unique RSSVM predictions overlap with predicted transcription terminators or attenuators. The 106 putative conserved terminators and the 123 predicted attenuators overlap in 74 orthologous UTR sets, which are more likely to contain real terminators. RSSVM, RNAz and QRNA identified 46 (71%), 30 (46%) and 23 (35%) of the common transcription terminators/attenuators, respectively, indicating that RSSVM has better sensitivity than RNAz and QRNA in finding putative conserved RNA motifs.

#### Predictions with literature support

We examined the leading genes of all the 166 operons with predicted regulatory RNAs by RSSVM (Table S2). 40 of the leading genes in these operons are hypothetical proteins that lack annotations. Of the 17 5′-UTRs containing predicted RNA motifs whose structure match known ncRNAs from Rfam, 5 of them encode hypothetical proteins. For the remaining 114 genes that have annotations and whose structures do not match ncRNAs from Rfam, we searched the literature for additional supporting evidence for our predictions. Table 3 lists 21 5′-UTRs which contain predicted RNA regulatory motifs that have been either identified or proposed from previous studies, such as *ilvG*, *ilvI*, *leuA*, *rpoB*, *rpsL*, *rplU*, *aspS*, *glnS*, *flgB* and *fliE*, or whose downstream genes or orthologs have been shown to be post-transcriptionally regulated in *S. oneidensis* or other bacterial species but without proposed regulatory RNA structures, such as *ldhA*, *aroH*, *adhE*, *ahpC*, *SO1769*, *pflB*, *SO3896*, *speA*, *secE* and *aroF*. RNAz and QRNA identified 11 and 7 of these 21 motifs with supporting literature evidence, respectively.

**Table 3:** Predicted regulatory RNAs that have supporting literature evidence.

Rank^a^	GI	RSSVM^b^	RNAz^b^	QRNA (Q) Terminator (T) Attenuator (A)	Gene Name	Gene Product	Knowledge of Regulation	Reference
5	**24350784**	**1.000**	**0.998**	Q T -	ilvG	acetolactate synthase II, large subunit	Leader peptide, and transcription attenuator	[48]
17	**24346570**	**1.000**	**0.507**	- T A	ldhA	D-lactate dehydrogenase	Possible post-transcriptional effect	[56]
23	**24348431**	**1.000**	0.105	- T -	aspS	aspartyl-tRNA synthetase	tRNA synthetase leader	
25	**24348233**	**1.000**	**0.904**	- - -	ilvI	acetolactate synthase III, large subunit	Leader peptide, and transcription attenuator	[48]
26	**24349427**	**1.000**	**0.862**	- - -	flgB	flagellar basal-body rod protein FlgB	Putative GEMM element	[20]
27	**24348700**	**1.000**	0.241	- - -	aroH	phospho-2-dehydro-3-deoxyheptonate aldolase, trp-sensitive	Possible transcription termination	[57]
35	**24350656**	**1.000**	**0.998**	Q T A	leuA	2-isopropylmalate synthase	Leader peptide, and transcription attenuator	[48]
41	**24345882**	**1.000**	0.094	- - -	pdhR	pyruvate dehydrogenase complex repressor	PdhR-box in *E. coli*	[58]
52	**24348056**	**1.000**	**0.893**	- T -	adhE	aldehyde-alcohol dehydrogenase	Stem-loop for occupying RBS in *E. coli*	[59]
55	**24346560**	**1.000**	0.196	- - -	ahpC	Alkyl hydroperoxide reductase, C subunit	Post-transcriptionally regulated by CsrA in *Helicobacter pylori*	[60]
63	**24347612**	**1.000**	0.012	Q T A	glnS	glutaminyl-tRNA synthetase	tRNA synthetase leader	[61]
83	**24347590**	**0.998**	0.451	- T A	SO1769	glutamate decarboxylase, putative	Possible post-transcriptional regulation in S. oneidensis	[47]
88	**24345631**	**0.998**	**0.999**	Q T -	rpoB	DNA-directed RNA polymerase, beta subunit	Transcriptional attenuation	[62],[63]
91	**24345625**	**0.998**	**1.000**	- - -	rplJ	ribosomal protein L10	Ribosomal protein leader	Rfam
105	**24349015**	**0.994**	0.274	Q T -	pflB	formate acetyltransferase	Possible post-transcriptional regulation	[64]
106	**24350214**	**0.994**	**0.688**	- - -	SO3896	Outer membrane porin, putative	Post-transcriptional regulation in S. oneidensis	[65]
109	**24345633**	**0.992**	**0.988**	Q T A	rpsL	ribosomal protein S12	Ribosomal protein leader	[32]
112	**24349403**	**0.990**	0.179	- T -	fliE	flagellar hook-basal body complex protein FliE	Putative GEMM element	[20]
124	**24345621**	**0.987**	0.108	- T -	secE	preprotein translocase, SecE subunit	RNaseIII sites in the leader sequence of SecE in E. coli	[66]
147	**24347716**	**0.972**	0.456	- T -	speA	biosynthetic arginine decarboxylase	Possible post-transcriptional regulation in S. oneidensis	[47]
161	**24347079**	**0.958**	**0.524**	Q - -	aroF	phospho-2-dehydro-3-deoxyheptonate aldolase, tyr-sensitive	Attenuator sensing tyr-tRNA	[67]
163	**24349925**	**0.956**	0.102	- - -	rplU	ribosomal protein L21	Ribosomal protein leader	[32]

a, b same as those in Table 2.

One class of our predicted RNA motifs correspond to known RNA regulatory motifs upstream of the operons involved in amino acid and vitamin biosynthesis, including *ilvG* and *ilvI* for isoleucine, *leuA* for leucine, *pheA* for phenylalanine, *tryE* for tryptophan, *thiC* for thiamin, *hisG* for histidine, *lysC* for lysine, and *aroH* and *aroF* for aromatic amino acids. One common regulatory mechanism for some of these operons, such as *ilvG*, *leuA*, *pheA*, *tryE* and *hisG*, is that the 5′-UTR of the mRNA contains a transcription terminator and a short sequence that encodes a leader peptide enriched with amino acids that are the synthesized products of the downstream genes. The translation of the leader peptide may sense the concentration of the amino-acid charged tRNAs in the cell. When the charged tRNA is abundant, the leader peptide can be translated successfully and a stable terminator structure is formed, blocking the transcription of the downstream genes. But during the shortage of the amino acid charged tRNA, the translation of the leader peptide is stalled and the terminator structure is opened, allowing the transcription of the downstream genes. Another regulatory mechanism for some of the operons involved in biosynthesis pathways, such as for *thiC* and *lysC*, is the riboswitch, in which the RNA structure can bind small metabolites, the product of the downstream genes, and stabilize a terminator structure which will shut down the transcription or translation of the downstream genes.

Another class of our predicted RNA motifs are located in the operons encoding ribosomal proteins and tRNA synthetases, such as *rpsB* (encoding ribosomal protein S2), *rplJ* (L10), *rpsL* (S12), *rplJ* (L21), *glnS* (glutaminyl-tRNA synthetase) and *aspS* (aspartyl-tRNA synthetase). A large number of the operons in these functional categories are known to be regulated post-transcriptionally by RNA structural elements [45],[46], and the enrichment of our predictions in these functional categories demonstrates the effectiveness of RSSVM.

In our predictions, some genes have been known to be regulated at the transcriptional level through binding of transcription factors (TF) to their palindromic DNA binding sites, such as *pdhR* (ranked 41), *metJ* and *metB* (ranked 72 and 89, sharing the same intergenic region). The palindromic TF binding sites contained in the promoters of these genes are usually long and have complementary mutations in the alignments. In a previous comparative genomics study [41], we identified 189 unique putative TF binding palindromic DNA motifs that are not only conserved across multiple *Shewanella* species, but also shared by multiple genes in *S. oneidensis*. 62 of these palindromic sites match known transcription factor binding motifs, and their corresponding regulated target genes also show significant functional enrichment or expression coherence, indicating that these predictions are likely to be real. 231 of all the 1002 UTRs here studied contain predicted palindromic DNA motifs that have at least one type of supporting evidence, and 38 of them are in the 166 top predictions by RSSVM. There is no significant enrichment (hypergeometric *p*-value = 0.57) for these putative palindromic sites in the RSSVM predictions, suggesting that the majority of the predictions by RSSVM are RNA structural motifs instead of palindromic TF binding sites. However, we cannot rule out the possibility that some genes may be regulated at both the transcriptional level (through TF DNA binding sites) and post-transcriptional level (through RNA regulatory motifs in mRNAs). Knowing the 5′ end of the mRNA would help resolve whether the predicted structure is within the mRNA, but that information is not currently available for most genes.

#### Putative candidates for novel regulatory RNA motifs

Besides the known RNA motifs, our predictions also include some interesting candidate novel motifs. One interesting example is for gene *SO3896*, which encodes an outer membrane porin, called *Omp35*. A previous study (Maier and Myers 2004) showed that the mRNA levels of *Omp35* in aerobic and anaerobic conditions are almost the same, but in anaerobic conditions the cell has 7-fold more *Omp35* protein than it does in aerobic condition, strongly suggesting that *Omp35* is regulated post-transcriptionally. In *E. coli*, some outer membrane porins, such as *OmpA* or *OmpW*, are regulated by antisense small RNAs (sRNA), *micA* and *rybB*. But these sRNAs are not found in *S. oneidensis*, suggesting that *S. oneidensis* may have a different regulatory mechanism for its outer membrane porins, one possibility being that regulatory RNA elements in the 5′-UTRs might be involved. Another example is for genes *SO1769* and *SpeA*, both of which are found to be regulated post-transcriptionally in *S. oneidensis* [47] and both contain predicted terminator structures in their 5′-UTRs from our study. It will be interesting to experimentally examine whether the putative terminator structures are actually involved in the regulation of these genes.

### Transcription Attenuator in the *LeuA* Operon

**Figure 5:**
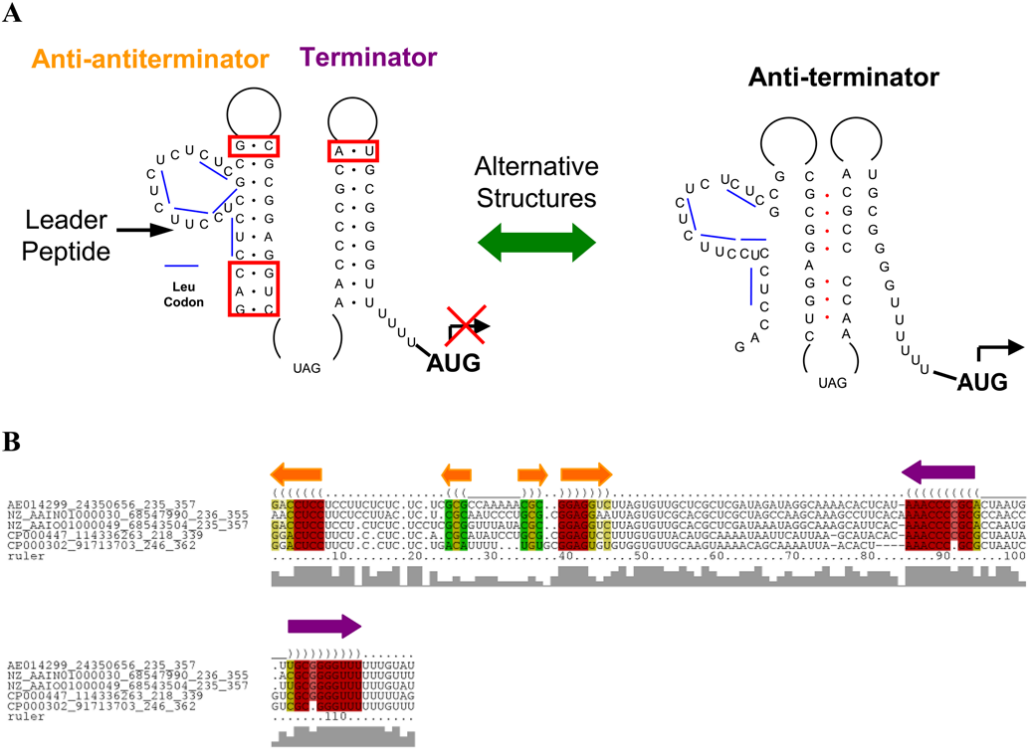
The predicted transcription terminator and anti-terminator structures of the *LeuA* operon in *Shewanella*. (A) Alternative terminator and anti-terminator stem-loop structures improved on the previously proposed structures. Base pairs in the red boxes are the positions where compensatory mutations are observed; blue lines are leucine codons enriched in the leader peptide coding region. (B) Structural alignment of the anti-antiterminator and terminator structure in five *Shewanella* species. The orange arrows correspond to the anti-antiterminator stem and the violet arrows correspond to the terminator stem. Colored columns represent aligned positions within the stems: red and pink colors represent conserved base pairings, and yellow and green colors represent base pairings with covariant mutations.

We use the predicted regulatory RNA motif in front of the *LeuA* operon as an example to illustrate detailed analysis of the predicted RNA structures. The predicted alternative structures in the 5′-UTR of the *LeuA* operon are shown in Figure 5A. Our predicted alternative structures match the majority of the previously proposed structures [48], including the terminator stem and the anti-terminator stem. Our predicted attenuator structure also includes an additional anti-antiterminator stem in front of the terminator stem. This anti-antiterminator is formed by part of the sequence encoding the leader peptide and half of the anti-terminator stem. The formation of the anti-antiterminator may halt the RNA polymerase, which pauses the transcription and allows translation of the leader peptide to start [8]. During the translation of the leader peptide, the anti-antiterminator stem is opened by the translation machinery and the paused RNA polymerase is able to resume transcription. When tRNA^Leu^ is adequate, the leader peptide can be successfully translated, releasing the ribosome at the stop codon of the leader peptide, and the reformation of the anti-antiterminator stem keeps the terminator structure intact which constitutively shuts down the transcription of the downstream genes. When the concentration of tRNA^Leu^ is low in the cell, the ribosome is stalled at the region enriched with leucine codons and the anti-antiterminator stem stays opened, which enables the formation of the anti-terminator and prevents the formation of the terminator stem, allowing transcription of the downstream genes. In the structural alignment of the predicted *LeuA* terminator motifs in the five *Shewanella* species (Figure 5B), we observed complementary mutations, which provide extra confidence to support the proposed anti-antiterminator structure.

## Discussion

In this paper, we present a new program, RSSVM, based on support vector machines for identifying putative *cis-*regulatory RNA motifs using the common secondary structures and structural alignments generated by RNA Sampler. By sequentially predicting common RNA secondary structures and alignments from orthologous UTRs and identifying putative RNA regulatory motifs based on the predicted structures and alignments, the combination of RNA Sampler and RSSVM provides a new, fast and efficient pipeline for large-scale searching of RNA regulatory motifs conserved in multiple related species. We applied this strategy to five *Shewanella* genomes and identified putative conserved *cis-*regulatory RNA motifs on the genome scale. From 1002 orthologous 5′-UTR sets, we successfully identified 166 5′-UTRs that contain putative regulatory RNA motifs, including 17 of 19 known RNA motifs from Rfam, additional 21 motifs with supporting literature evidence, 72 motifs that overlap with predicted transcription attenuators/terminators, and other novel predicted regulatory RNA motifs. The fact that a large fraction of our predictions are supported by published reports or overlap with predictions by RNAz, QRNA and transcription attenuator/terminator predictors suggests that many of our new predictions are likely to be real, although experimental validation will be needed.

Comparing to other RNA motif identification tools, such as RNAz, Dynalign+LIBSVM and QRNA, RSSVM is more sensitive in detecting functional RNAs at the same FPR, especially on sequences of low identities. The more sensitive performance of RSSVM, compared to that of RNAz and Dynalign+LIBSVM, may be attributed to the following three improvements in the SVM model: first, the common structures and alignments are generated by RNA Sampler, which provides more accurate structure predictions, does not require sequence alignments as input and works well on sequences of low identities; second, more distinctive features are used to represent the common RNA structures and alignments; third, the SVM model is trained with more universal functional RNA structures that cover a large number of RNA motif/gene families and a wide range of sequence identities. We tested a few alternative SVM models which have only one or two of these improvements, such as a modified RNAz that is re-trained using the same training sets for RSSVM, and a modified RNAz that is re-trained using the same training sets for RSSVM and that uses RNA Sampler's structural alignments instead of ClustalW alignments as input. We observed that the sensitivities of these SVM models on all test sets and on test sets with low identities were higher than those of RNAz and similar to those of RSSVM when loose FPRs were allowed, and their sensitivities were gradually improved in the stringent FPR range (FPR<0.02) by adding one improvement at a time (Figure S2). For a tool designed for genome-wide motif prediction, it is essential to keep the FPR as low as possible while achieving a high sensitivity, as with a large number of data sets, low FPRs are always preferred to avoid bringing too many false positives in the predictions. RSSVM, which combines all the three improvements, gives much better sensitivities than other SVM models in the low FPR range (FPR<0.02), suggesting that RSSVM is well qualified for using in large scale predictions.

RNA Sampler and RSSVM run reasonably fast for genome-wide scan of regulatory RNAs. On average, it takes RNA Sampler 125 seconds on a single CPU workstation to predict the common structure of a set of 5 RNA sequences of an average of 150 nt in length. For a project with the similar size of the *Shewanella* study (1000 orthologous UTR sets, 5 species, 3 scanning windows of 150 nt, one shuffled set for each UTR set), it only takes RSSVM about 200 hours on a single CPU machine to finish the genome-wide scan of RNA motifs. The entire process can be easily run in parallel on multiple-CPU Linux clusters, allowing the whole genome prediction to be done in hours or less. We recommend that users run RNAz as well, since RNAz has better performance on sequences of high identity, which is complementary to the optimum performance of RSSVM on low-identity sequences. Moreover, consensus predictions by both approaches may provide extra confidence in the prediction quality. RSSVM is more advantageous than Dynalign+LIBSVM and QRNA in that RSSVM can take input of multiple sequences, which would provide more information on sequence conservation and complementary mutations than two sequences. Recently, Yao et al. [32] built a pipeline based on CMfinder and successfully identified several new RNA motifs. The major advantages of the CMfinder pipeline lie in its relaxation on the requirement for sequence conservation and integration of motif inference in the genome-wide search. It builds in a scanning procedure which can conveniently look for new instances of motifs in other genomes. However, one major issue of the CMfinder pipeline is that it uses a heuristic composite scoring function to sort all its predictions without giving a clear significance cutoff for confident calls for positive predictions. Also, the pipeline of CMfinder runs considerably slower than RSSVM. Using CMfinder for refinement and new instance finding on interesting predictions from a search by RSSVM may be more efficient and rewarding for users.

The RNA classification of RSSVM is based on the common structures generated by RNA Sampler. These predicted common structures provide preliminary hints for the putative structures associated to the regulatory functions. As demonstrated in the RNA motifs for *LeuA*, we can infer function and mechanism of the RNA motif from its structure. Although we cannot guarantee that the predicted structures are correct and perfectly match the real structures, they often indicate strong structural conservation information in the potential regulatory regions, which leads to sensitive detection of RNA motifs. Users can use Mfold [49] or other programs to refold the identified regions to obtain sub-optimal structures, which may provide good candidates for alternative structures related to the function of the RNA motifs.

There is always a trade-off between sensitivity and specificity (1 – false positive rate) in computational predictions. Using looser cutoffs (lower *P*-values in our case) will help increase prediction sensitivities, but at the same time more false positives may appear in the predictions. As seen in the ROC curves generated on the test sets (Figure 1), RSSVM keep high sensitivities even at very stringent FPRs, which makes it a good tool to be used in the genome-wide scan of RNA motifs. In the *Shewanella* RNA motif study, although we used a very stringent *P*-value cutoff, which corresponded to FPR<0.01 on the test sets, we still discovered most of the known RNA motifs. However, we noticed that some of the known RNA motifs were scored slightly below this cutoff. Users may consider lowering the cutoff in their own studies depending upon the tolerance to false positives.

Application of RSSVM to find RNA regulatory motifs/genes is not limited to the *Shewanella* genomes. This approach is fully transferable to other bacterial genomes, or in fact to any set of orthologous RNA segments that are suspected of containing conserved secondary structure motifs. We conducted some pilot tests on the classification performance of RSSVM on RNA sequences from eukaryotic genomes. Without retraining it, we ran RSSVM on 4087 sets of real RNA sequences (positive eukaryotic test sets) from 372 eukaryotic RNA motif families from Rfam and the same number of shuffled sequence sets (negative eukaryotic test sets). Each sequence set contains 3–6 sequences whose average sequence identities ranges from 20% to 100%. The ROC curves on these test sequences are shown in Figure S3. Encouragingly, RSSVM performs well in this test: at the FPR of 0.02, RSSVM gives a good prediction sensitivity of 0.5, lower than it does on the prokaryotic test sets (0.72 at FPR of 0.02) as expected (Dataset S2). Consistent with the prokaryotic tests, RSSVM becomes more sensitive than RNAz when FPR is greater than 0.02. Again, RSSVM performs much better on sequence sets whose identities are lower than 70%, with the overall prediction sensitivity jumping to 0.6 at the FPR of 0.02. On these low-identity sequences, RSSVM starts to outperform RNAz from a very low FPR of 0.005. These results suggest that the RSSVM model trained on prokaryotic RNAs can also be used to search for RNA motifs in eukaryotic sequences. It also verifies that RSSVM can find novel RNA motifs that are distinctive from those in the training sets. By re-training RSSVM on sequence sets from eukaryotic RNA families, its performance may be further improved.

To better serve the *Shewanella* research community and research groups who are interested in RNA regulatory motifs or post-transcriptionally regulation, we made the RSSVM software, predictions and comprehensive analysis results available online at http://ural.wustl.edu/resources.html#RSSVM.

## Methods

### Common RNA Secondary Structure Prediction

We use the program, RNA Sampler [27], to predict common RNA secondary structures and generate structural alignments in homologous sequences. RNA Sampler is a probabilistic sampling algorithm that was recently developed by our group. In previous tests [27]–[29], RNA Sampler outperformed other leading algorithms for similar purposes on sequences of a wide range of identities. In this study, the default parameters of RNA Sampler, *S* = 75 (structure sample size) and *i* = 15 (iterations), were used in all predictions. Although RNA Sampler is able to predict RNA secondary structures with pseudoknots, we opted to not allow pseudoknots in this study.

### The RNA Classifier Based on Support Vector Machines

Support Vector Machines (SVM) are supervised learning methods widely used for classification and regression. In these methods, labeled data are represented by vectors that are defined by various features, and support vector machines map the feature vectors to a higher dimensional space and construct a maximal separating hyperplane to classify the input data into binary categories. SVM has been used in previous studies [34],[35] to distinguish regulatory RNA secondary structures from random RNA structures. In such methods, the RNA secondary structures or structural alignments of homologous RNAs are represented by a set of predefined features, and the SVM maps the vectors defined by these features to a high-dimensional space. By training on the RNA structures or structural alignments of known functional and random RNAs, SVM is able to maximally separate these two groups of RNAs. Then for any unknown RNA secondary structure, SVM can classify it as either functional or random.

We developed a new SVM classifier for detecting regulatory RNAs. Our SVM classifier differs from the previous ones in three major aspects: first, the recently developed new program, RNA Sampler, is used to predict common RNA secondary structures and structural alignments on any set of homologous RNA sequence, and feature vectors based on such predictions are used to build the SVM classifier; second, a different set of feature parameters are used to represent the common RNA structures and structural alignments; third, the SVM classifier is trained on a larger number of various bacterial RNA gene and motif families that cover a wider range of sequence lengths and identities than previous studies [34],[35].

#### Training sets and testing sets

To train the SVM classifier, both positive and negative training sets are needed. We use sequences of 112 known bacterial regulatory RNA families retrieved from the Rfam database [37] to generate the positive training sets. Each positive sequence set contains 3, 4, 5 or 6 sequences randomly selected from the Rfam seed alignment of each RNA family. The lengths of the selected sequences are between 50 and 400 nt and the maximum length difference between any two sequences in a set is less than 50 nt. These training sets contain non-redundant structural alignments of different sequence numbers and evenly cover a wide range of sequence identities (20–90%). For each positive training set, a corresponding negative training set is generated by randomly shuffling the Rfam structural alignment of the positive set, destroying the common RNA structure but preserving base composition, overall conservation, local conservation pattern and gap patterns of the original alignment [34].

In total, we generated 8335 positive and 8335 negative training sequence sets. Using a similar procedure, we generated 1686 positive and 1686 negative test sequence sets that are not identical to any training set. The distributions of the sizes and average pairwise sequence identities of the training and test sets are shown in Figure S4.

#### Features to represent the common RNA structure and structural alignment

In our SVM classifier, we use six features to represent the common RNA secondary structure and structural alignment. These features are: (1) The mean minimum free energy (MFE) *z* score of all sequences sharing the common structure (*Z*) [34]. The MFE *z* score measures the thermodynamic stability of a sequence by comparing the MFE of the sequence to the MFE distribution of random sequences of similar length and base composition. (2) The structure conservation index (*SCI*) of the common structure, measuring the conserved structure information contained in the structural alignment. It is defined as *SCI = E_A_/Ē*, where *E_A_* is the consensus MFE of the common structure and *Ē* is the average MFE of all sequences [34]. (3) The average information content of stems (*I*), measuring the sequence conservation of the stems in a structural alignment [50]. (4) The average mutual information of the stems in the structural alignment (*M*), measuring the overall covariation of the complementary columns in the stems [51]. (5) The average pairwise sequence identity. (6) The number of sequences. These features represent different characteristics of the common structure and structural alignment, and each feature alone is not able to effectively distinguish real regulatory RNA structures from random common RNA structures.

#### Training of the SVM classifier

On each training sequence set, we ran RNA Sampler to generate the common structure and structural alignment and calculated the values of the six features described above. We implemented the SVM classifier for regulatory RNA structure detection using the core program LIBSVM [52]. Our SVM classifier uses a radial-basis-function (RBF) kernel. Prior to input to LIBSVM, values of all features are scaled to the range of [−1, 1]. Training an SVM model finds the best parameters of the penalty of the error term (*c*) and the value of gamma of the RBF (*g*) to define a hyper-dimensional space that gives maximal separation of the positive (real RNA motifs) and negative training data (shuffled RNA motifs). We did a grid search to find the best combination of *c* and *g* within certain ranges (*c∈*[2^−10^, 2^15^], *g∈*[2^−10^, 2^15^]). To avoid overfitting the training data, we employed a 5-fold cross-validation, in which the whole training sets were divided into 5 subsets of equal size and sequentially one subset was tested using the classifier trained on the remaining 4 subsets. Because each training set was predicted once, the accuracy of the cross-validation was the percentage of data which were correctly classified. We determined that the combination of *c* = 2^10^ and *g* = 2^−3.5^ gave one of the best classification results and fewer false positive predictions. Thus we used these *c* and *g* parameters to train the SVM classifier on the whole training sets to obtain the final classification model. The option of “*−b* 1” was used to report confidence probability of classification (*P*-value).

#### Testing of the SVM classifier

We predicted the common structures and structure alignments for all positive and negative test sets using RNA Sampler and classified the structures with the final SVM model. We also compared the performance of our SVM classifier on these test sets with that of other leading software for RNA motif identification, including RNAz [34], Dynalign+LIBSVM [35] and QRNA[33]. RNAz is also an SVM based RNA motif classifier, which takes sequence alignments as input and uses RNAalifold as the core algorithm for common structure prediction. It was trained on sequences with high identities (≥50%) using the features of MFE *z* scores and *SCI* scores [34]. Dynalign+LIBSVM is another SVM based RNA motif identifier. It takes two unaligned sequences as input and uses Dynalign as the core algorithm to predict common structures. It was trained only on tRNAs and 5S rRNAs using the feature of free energy change [35]. QRNA classifies a pairwise sequence alignment by the posterior probabilities of three probabilistic models, “RNA”, “Coding”, or “Null” (position independent). The differences between RSSVM, RNAz, Dynalign+LIBSVM and QRNA are summarized in Table S3. We used ClustalW [53] to generate sequence alignments for RNAz. For each test set, we split the ClustalW alignment to all possible pairwise sequence alignments as input for QRNA. Dynalign+LIBSVM was tested on all unique pairs of sequences for each test set. RNAz, Dynalign+LIBSVM and QRNA were run with default parameters.

### Searching Regulatory RNAs in *Shewanella* Genomes

With the RNA secondary structure prediction algorithm, RNA Sampler, and the RNA motif identification algorithm, RSSVM, we can search putative regulatory RNA structural motifs from any orthologous RNA sequence set. As shown in the flow chart in Figure S5, we first retrieve orthologous RNA sequences from multiple related species, and then use RNA Sampler to predict common RNA secondary structures and structural alignments of these orthologous RNA sequences. Next, RSSVM takes in the output from RNA Sampler and identifies those containing putative RNA motifs/genes. Finally, we evaluate the prediction results by comparing to known RNA motifs or searching for supporting evidence. We applied the combination of RNA Sampler and RSSVM to multiple *Shewanella* genomes for genome-wide regulatory RNA discovery.

#### Retrieval of mRNA leader sequences of orthologous genes

The genomic sequences of *Shewanella oneidensis MR-1, S. denitrificans OS217, S. frigidimarina NCIMB 400, S. amazonensis SB2B* and *S. baltica OS155* were downloaded from the NCBI Genebank database.

Since most of the known bacterial regulatory motifs are located in the mRNA leader sequences of the regulated transcription units (operons), we focused on finding conserved RNA regulatory motifs in the 5′-UTRs of orthologous transcription units (TU). Because our knowledge of the operon structures in *S. oneidensis* is limited, we assumed that two adjacent genes in the same orientation whose intergenic distance is less than 40 nt are in the same transcription unit [54]. For each TU in *S. oneidensis*, we identified its first gene and this gene's orthologs in the other four *Shewanella* species, and then retrieved 120∼250 nt 5′-UTR sequence upstream and 20 nt downstream of the translation start codons of these genes as one set of orthologous sequences for RNA secondary structure search. We identified orthologous genes by comparing all protein sequences between *S. oneidensis* (the anchor genome) and the other species using the WU-BLAST program (version 2.0, Gish, W., http://blast.wustl.edu) [55]. Two genes were considered orthologous if all of the following conditions were met: (i) their protein sequences were reciprocal best BLASTP hits between the two genomes; (ii) the BLASTP E-value was lower than 1.0×10^−10^; and (iii) the BLASTP alignment covered ≥60% of the length of at least one sequence.

In total we obtained 1002 sets of orthologous mRNA leader sequences from the five *Shewanella* species. The mRNA leader sequences obtained had a high AU content, with a base composition of A 31%, U 29%, C 19% and G 21%.

#### Searching for regulatory RNA structures

We scanned each orthologous UTR sequence set in three overlapping windows, −250∼−100, −200∼−50, and −150∼20 (1 corresponds to the translation start site). We first predicted the common RNA structure for each window and generated corresponding structural alignment using RNA Sampler, and then provided the RNA Sampler output to the SVM classifier to predict whether the window contains a regulatory RNA structure. We also aligned sequences in the same window using ClustalW and applied RNAz and QRNA to predict the existence of RNA motifs. Because QRNA only takes two-sequence alignments as input, for each aligned sequence window, we split the multiple-sequence ClustalW alignment into pairwise alignments, each consisting of the sequence from the anchor species and a sequence from another species. We call that QRNA detected the RNA motifs in the sequence window only if at least two of all the pairwise alignments were predicted to contain an RNA motif by QRNA.

## Supporting Information

Dataset S1 Raw Data for ROC curves in Figure 1.
(0.57 MB XLS)

Dataset S2 Raw Data for ROC curves in Figure S3.
(0.50 MB XLS)

Figure S1 Cumulative distribution of sequence identities of the sequence sets with predicted regulatory RNAs by RSSVM and/or RNAz.
(0.01 MB PDF)

Figure S2 The Receiver Operating Characteristic (ROC) curves of RSSVM, RNAz, retrained RNAz on ClustalW alignments, and retrained RNAz on RNA Sampler alignments. (A) On all test sets. (B) On test sets with sequence identities lower than 70%. We retrained RNAz using the same training sets for RSSVM.
(0.02 MB PDF)

Figure S3 The Receiver Operating Characteristic (ROC) curves of RSSVM and RNAz on real and shuffled sequence sets of eukaryotic RNAs from Rfam. The curves of both programs on all test sets (sequence identities range between 20–100%) and on test sets of low identities (<70%) are drawn separately.
(0.01 MB PDF)

Figure S4 Distribution of sequence identities and size of the sequence sets studied. (A) Training sets. (B) Test sets. (C) *Shewanella* sequence sets.
(0.01 MB PDF)

Figure S5 Flow chart of the genome-wide identification of RNA regulatory motifs/genes using RNA Sampler and RSSVM.
(0.01 MB PDF)

Table S1 The prediction sensitivities and false positive rates of RSSVM, RNAz, Dynalign+LIBSVM and QRNA on test sets with different sequence identities. Different P-value cutoffs were used to fairly compare prediction sensitivities of different SVM models at the same FPR level. Numbers in bold fonts are the best results given by all the programs for an identity range.
(0.01 MB PDF)

Table S2 Top 166 predicted regulatory RNAs by RSSVM.
(0.06 MB PDF)

Table S3 Comparisons between different RNA motif identification algorithms.
(0.01 MB PDF)

## References

[1] Gesteland RF, Cech T, Atkins JF (1999). The RNA World: The Nature of Modern RNA Suggests a Prebiotic RNA.

[2] Eddy SR (2001). Non-coding RNA genes and the modern RNA world.. Nat Rev Genet.

[3] Steitz TA (2008). A structural understanding of the dynamic ribosome machine.. Nat Rev Mol Cell Biol.

[4] Doherty EA, Doudna JA (2001). Ribozyme structures and mechanisms.. Annu Rev Biophys Biomol Struct.

[5] Fedor MJ, Williamson JR (2005). The catalytic diversity of RNAs.. Nat Rev Mol Cell Biol.

[6] Gottesman S (2002). Stealth regulation: biological circuits with small RNA switches.. Genes Dev.

[7] He L, Hannon GJ (2004). MicroRNAs: small RNAs with a big role in gene regulation.. Nat Rev Genet.

[8] Henkin TM, Yanofsky C (2002). Regulation by transcription attenuation in bacteria: how RNA provides instructions for transcription termination/antitermination decisions.. Bioessays.

[9] Vitreschak AG, Rodionov DA, Mironov AA, Gelfand MS (2004). Riboswitches: the oldest mechanism for the regulation of gene expression?. Trends Genet.

[10] Blount KF, Breaker RR (2006). Riboswitches as antibacterial drug targets.. Nat Biotechnol.

[11] Bumcrot D, Manoharan M, Koteliansky V, Sah DW (2006). RNAi therapeutics: a potential new class of pharmaceutical drugs.. Nat Chem Biol.

[12] Lavery KS, King TH (2003). Antisense and RNAi: powerful tools in drug target discovery and validation.. Curr Opin Drug Discov Devel.

[13] Stormo GD, Ji Y (2001). Do mRNAs act as direct sensors of small molecules to control their expression?. Proc Natl Acad Sci U S A.

[14] Batey RT (2006). Structures of regulatory elements in mRNAs.. Curr Opin Struct Biol.

[15] Winkler WC (2005). Riboswitches and the role of noncoding RNAs in bacterial metabolic control.. Curr Opin Chem Biol.

[16] Nogueira T, Springer M (2000). Post-transcriptional control by global regulators of gene expression in bacteria.. Curr Opin Microbiol.

[17] Mandal M, Boese B, Barrick JE, Winkler WC, Breaker RR (2003). Riboswitches control fundamental biochemical pathways in Bacillus subtilis and other bacteria.. Cell.

[18] Huttenhofer A (2006). RNomics: identification and function of small non-protein-coding RNAs in model organisms.. Cold Spring Harb Symp Quant Biol.

[19] McCutcheon JP, Eddy SR (2003). Computational identification of non-coding RNAs in Saccharomyces cerevisiae by comparative genomics.. Nucleic Acids Res.

[20] Weinberg Z, Barrick JE, Yao Z, Roth A, Kim JN (2007). Identification of 22 candidate structured RNAs in bacteria using the CMfinder comparative genomics pipeline.. Nucleic Acids Res.

[21] Hofacker IL, Fekete M, Stadler PF (2002). Secondary structure prediction for aligned RNA sequences.. J Mol Biol.

[22] Mathews DH, Turner DH (2002). Dynalign: an algorithm for finding the secondary structure common to two RNA sequences.. J Mol Biol.

[23] Ji Y, Xu X, Stormo GD (2004). A graph theoretical approach for predicting common RNA secondary structure motifs including pseudoknots in unaligned sequences.. Bioinformatics.

[24] Yao Z, Weinberg Z, Ruzzo WL (2006). CMfinder—a covariance model based RNA motif finding algorithm.. Bioinformatics.

[25] Gorodkin J, Heyer LJ, Stormo GD (1997). Finding the most significant common sequence and structure motifs in a set of RNA sequences.. Nucleic Acids Res.

[26] Torarinsson E, Havgaard JH, Gorodkin J (2007). Multiple structural alignment and clustering of RNA sequences.. Bioinformatics.

[27] Xu X, Ji Y, Stormo GD (2007). RNA Sampler: a new sampling based algorithm for common RNA secondary structure prediction and structural alignment.. Bioinformatics.

[28] Torarinsson E, Lindgreen S (2008). WAR: Webserver for aligning structural RNAs.. Nucleic Acids Res.

[29] Wilm A, Higgins DG, Notredame C (2008). R-Coffee: a method for multiple alignment of non-coding RNA.. Nucleic Acids Res.

[30] Workman C, Krogh A (1999). No evidence that mRNAs have lower folding free energies than random sequences with the same dinucleotide distribution.. Nucleic Acids Res.

[31] Rivas E, Eddy SR (2000). Secondary structure alone is generally not statistically significant for the detection of noncoding RNAs.. Bioinformatics.

[32] Yao Z, Barrick J, Weinberg Z, Neph S, Breaker R (2007). A computational pipeline for high- throughput discovery of cis-regulatory noncoding RNA in prokaryotes.. PLoS Comput Biol.

[33] Rivas E, Eddy SR (2001). Noncoding RNA gene detection using comparative sequence analysis.. BMC Bioinformatics.

[34] Washietl S, Hofacker IL, Stadler PF (2005). Fast and reliable prediction of noncoding RNAs.. Proc Natl Acad Sci U S A.

[35] Uzilov AV, Keegan JM, Mathews DH (2006). Detection of non-coding RNAs on the basis of predicted secondary structure formation free energy change.. BMC Bioinformatics.

[36] Hofacker IL (2007). RNA consensus structure prediction with RNAalifold.. Methods Mol Biol.

[37] Griffiths-Jones S, Moxon S, Marshall M, Khanna A, Eddy SR (2005). Rfam: annotating non-coding RNAs in complete genomes.. Nucleic Acids Res.

[38] Beliaev AS, Thompson DK, Fields MW, Wu L, Lies DP (2002). Microarray transcription profiling of a Shewanella oneidensis etrA mutant.. J Bacteriol.

[39] Bencheikh-Latmani R, Williams SM, Haucke L, Criddle CS, Wu L (2005). Global transcriptional profiling of Shewanella oneidensis MR-1 during Cr(VI) and U(VI) reduction.. Appl Environ Microbiol.

[40] Heidelberg JF, Paulsen IT, Nelson KE, Gaidos EJ, Nelson WC (2002). Genome sequence of the dissimilatory metal ion-reducing bacterium Shewanella oneidensis.. Nat Biotechnol.

[41] Liu J, Xu X, Stormo GD (2008). The cis-regulatory map of Shewanella genomes.. Nucleic Acids Res.

[42] Nawrocki EP, Eddy SR (2007). Query-dependent banding (QDB) for faster RNA similarity searches.. PLoS Comput Biol.

[43] Wan XF, Lin G, Xu D (2006). Rnall: an efficient algorithm for predicting RNA local secondary structural landscape in genomes.. J Bioinform Comput Biol.

[44] Merino E, Yanofsky C (2005). Transcription attenuation: a highly conserved regulatory strategy used by bacteria.. Trends Genet.

[45] Condon C, Grunberg-Manago M, Putzer H (1996). Aminoacyl-tRNA synthetase gene regulation in Bacillus subtilis.. Biochimie.

[46] Lindahl L, Zengel JM (1986). Ribosomal genes in Escherichia coli.. Annu Rev Genet.

[47] Yang Y, Harris DP, Luo F, Wu L, Parsons AB (2008). Characterization of the Shewanella oneidensis Fur gene: roles in iron and acid tolerance response.. BMC Genomics.

[48] Vitreschak AG, Lyubetskaya EV, Shirshin MA, Gelfand MS, Lyubetsky VA (2004). Attenuation regulation of amino acid biosynthetic operons in proteobacteria: comparative genomics analysis.. FEMS Microbiol Lett.

[49] Zuker M (2003). Mfold web server for nucleic acid folding and hybridization prediction.. Nucleic Acids Res.

[50] Stormo GD (2000). DNA binding sites: representation and discovery.. Bioinformatics.

[51] Gorodkin J, Heyer LJ, Brunak S, Stormo GD (1997). Displaying the information contents of structural RNA alignments: the structure logos.. Comput Appl Biosci.

[52] Chang C, Lin C (2001). LIBSVM: a library for support vector machines.. http://www.csie.ntu.edu.tw/~cjlin/libsvm.

[53] Thompson JD, Higgins DG, Gibson TJ (1994). CLUSTAL W: improving the sensitivity of progressive multiple sequence alignment through sequence weighting, position-specific gap penalties and weight matrix choice.. Nucleic Acids Res.

[54] Salgado H, Moreno-Hagelsieb G, Smith TF, Collado-Vides J (2000). Operons in Escherichia coli: genomic analyses and predictions.. Proc Natl Acad Sci U S A.

[55] Altschul SF, Gish W, Miller W, Myers EW, Lipman DJ (1990). Basic local alignment search tool.. J Mol Biol.

[56] Jiang GR, Nikolova S, Clark DP (2001). Regulation of the ldhA gene, encoding the fermentative lactate dehydrogenase of Escherichia coli.. Microbiology.

[57] Zurawski G, Gunsalus RP, Brown KD, Yanofsky C (1981). Structure and regulation of aroH, the structural gene for the tryptophan-repressible 3-deoxy-D-arabino-heptulosonic acid-7-phosphate synthetase of Escherichia coli.. J Mol Biol.

[58] Ogasawara H, Ishida Y, Yamada K, Yamamoto K, Ishihama A (2007). PdhR (pyruvate dehydrogenase complex regulator) controls the respiratory electron transport system in Escherichia coli.. J Bacteriol.

[59] Membrillo-Hernandez J, Lin EC (1999). Regulation of expression of the adhE gene, encoding ethanol oxidoreductase in Escherichia coli: transcription from a downstream promoter and regulation by fnr and RpoS.. J Bacteriol.

[60] Barnard FM, Loughlin MF, Fainberg HP, Messenger MP, Ussery DW (2004). Global regulation of virulence and the stress response by CsrA in the highly adapted human gastric pathogen Helicobacter pylori.. Mol Microbiol.

[61] Cheung AY, Watson L, Soll D (1985). Two control systems modulate the level of glutaminyl-tRNA synthetase in Escherichia coli.. J Bacteriol.

[62] Steward KL, Linn T (1992). Transcription frequency modulates the efficiency of an attenuator preceding the rpoBC RNA polymerase genes of Escherichia coli: possible autogenous control.. Nucleic Acids Res.

[63] Ishihama A, Fukuda R (1980). Autogenous and post-transcriptional regulation of RNA polymerase synthesis.. Mol Cell Biochem.

[64] Beliaev AS, Thompson DK, Khare T, Lim H, Brandt CC (2002). Gene and protein expression profiles of Shewanella oneidensis during anaerobic growth with different electron acceptors.. OMICS.

[65] Maier TM, Myers CR (2004). The outer membrane protein Omp35 affects the reduction of Fe(III), nitrate, and fumarate by Shewanella oneidensis MR-1.. BMC Microbiol.

[66] Chow J, Dennis PP (1994). Coupling between mRNA synthesis and mRNA stability in Escherichia coli.. Mol Microbiol.

[67] Camakaris H, Camakaris J, Pittard J (1980). Regulation of aromatic amino acid biosynthesis in Escherichia coli K-12: control of the aroF-tyrA operon in the absence of repression control.. J Bacteriol.

