# Supplementary material for: Discovering cis-Regulatory RNAs in Shewanella Genomes by Support Vector Machines
Source: PLoS Comput Biol. 2009 Apr 3;5(4):e1000338. doi: 10.1371/journal.pcbi.1000338 (PMC2659441; doi:10.1371/journal.pcbi.1000338)
Supplement: Figure S1 — Cumulative distribution of sequence identities of the sequence sets with predicted regulatory RNAs by RSSVM and/or RNAz. (0.01 MB PDF) [file pcbi.1000338.s003.pdf]

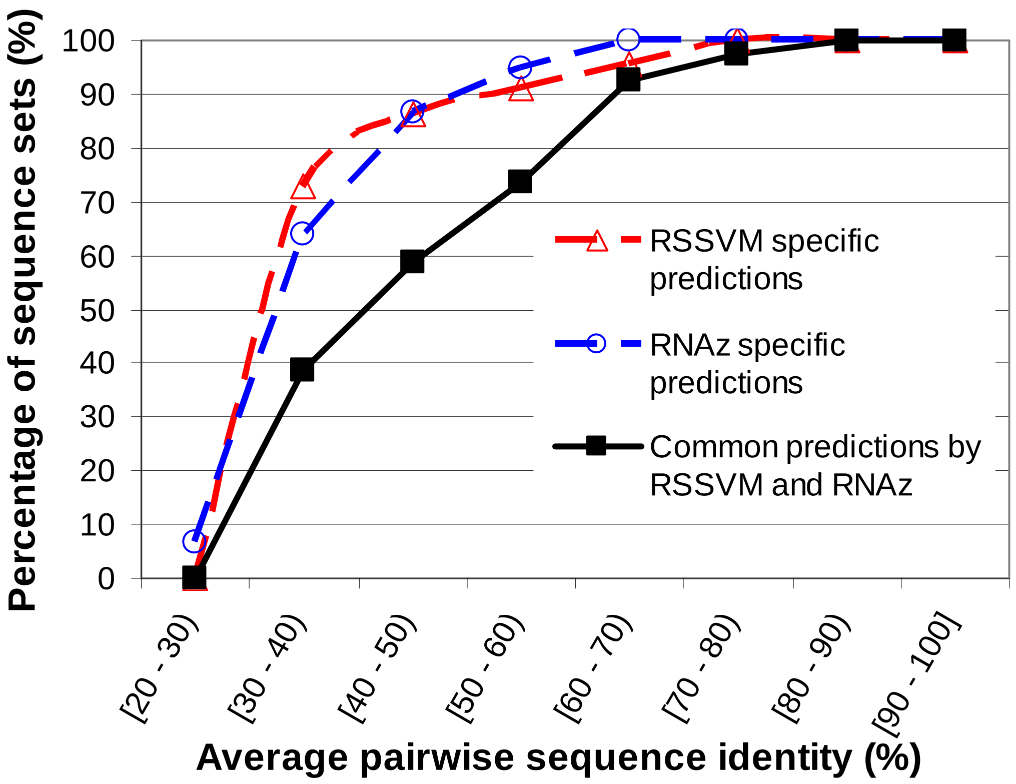


**Figure S1.** Cumulative distribution of sequence identities of the sequence sets with predicted regulatory RNAs by RSSVM and/or RNAz.
