# Supplementary material for: Discovering cis-Regulatory RNAs in Shewanella Genomes by Support Vector Machines
Source: PLoS Comput Biol. 2009 Apr 3;5(4):e1000338. doi: 10.1371/journal.pcbi.1000338 (PMC2659441; doi:10.1371/journal.pcbi.1000338)
Supplement: Figure S2 — The Receiver Operating Characteristic (ROC) curves of RSSVM, RNAz, retrained RNAz on ClustalW alignments, and retrained RNAz on RNA Sampler alignments. (A) On all test sets. (B) On test sets with sequence identities lower than 70%. We retrained RNAz using the same training sets for RSSVM. (0.02 MB PDF) [file pcbi.1000338.s004.pdf]

A.

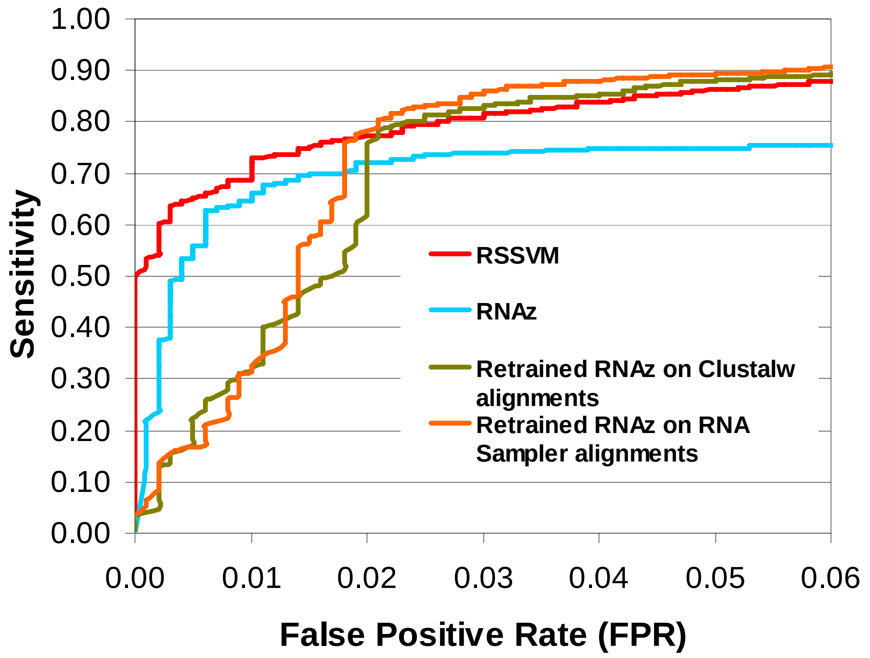

B.

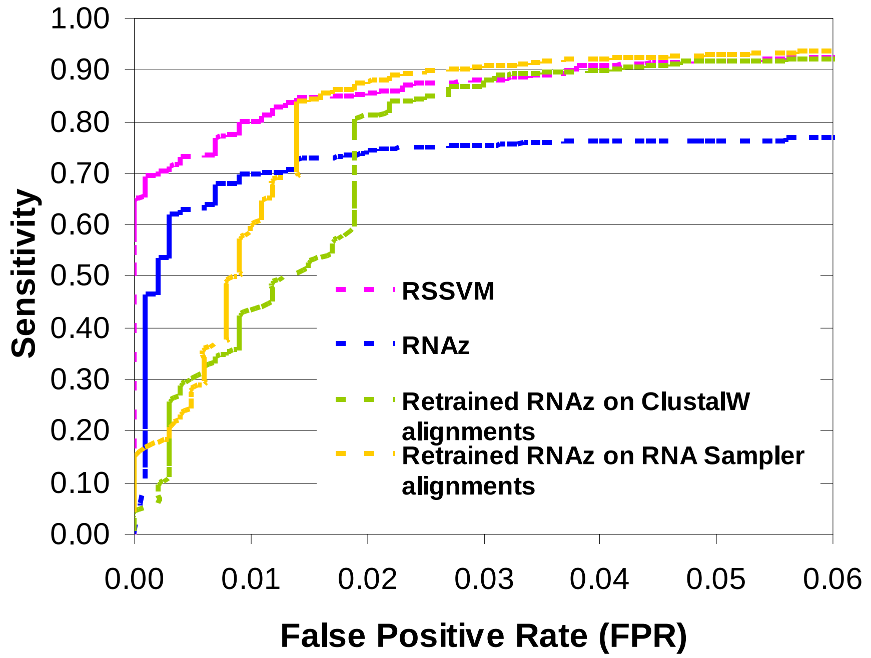

Figure S2. The Receiver Operating Characteristic (ROC) curves of RSSVM, RNAz, retrained RNAz on ClustalW alignments, and retrained RNAz on RNA Sampler alignments. A. on all test sets. B on test sets with sequence identities lower than 70%. We retrained RNAz using the same training sets for RSSVM.
