# Supplementary material for: Discovering cis-Regulatory RNAs in Shewanella Genomes by Support Vector Machines
Source: PLoS Comput Biol. 2009 Apr 3;5(4):e1000338. doi: 10.1371/journal.pcbi.1000338 (PMC2659441; doi:10.1371/journal.pcbi.1000338)
Supplement: Figure S3 — The Receiver Operating Characteristic (ROC) curves of RSSVM and RNAz on real and shuffled sequence sets of eukaryotic RNAs from Rfam. The curves of both programs on all test sets (sequence identities range between 20–100%) and on test sets of low identities (<70%) are drawn separately. (0.01 MB PDF) [file pcbi.1000338.s005.pdf]

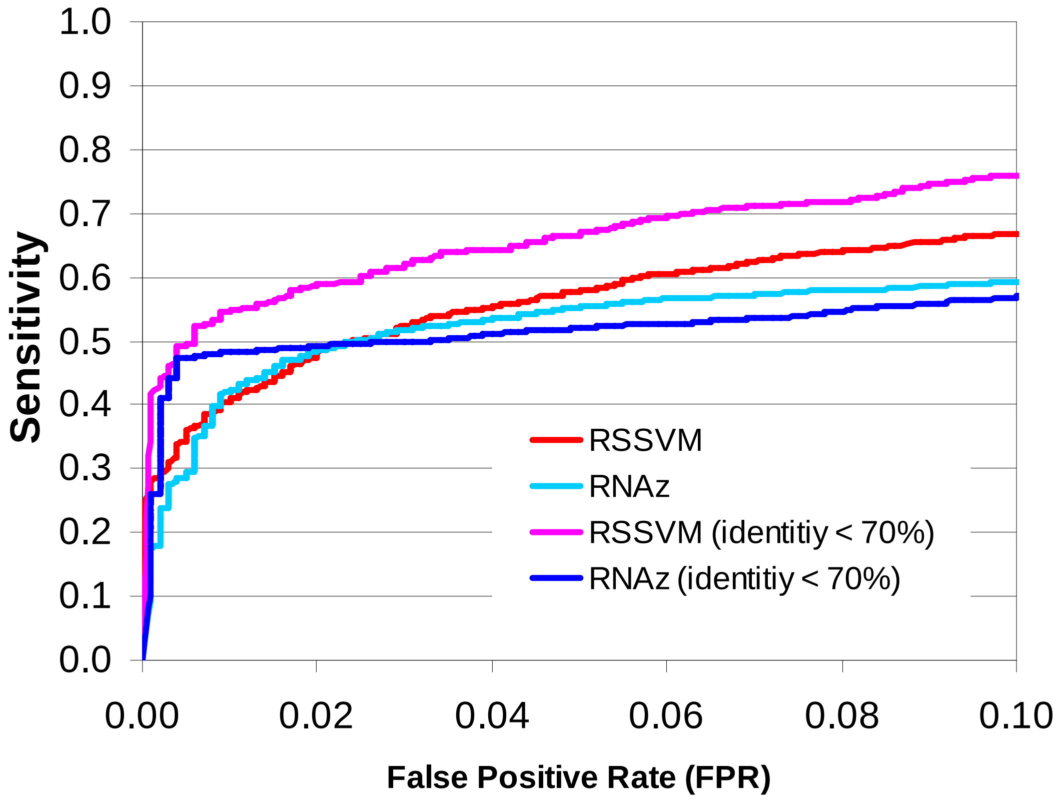

Figure S3. The Receiver Operating Characteristic (ROC) curves of RSSVM and RNAz on real and shuffled sequence sets of eukaryotic RNAs from Rfam. The curves of both programs on all test sets (sequence identities range between 20-100%) and on test sets of low identities (< 70%) are drawn separately.
