# Supplementary material for: Discovering cis-Regulatory RNAs in Shewanella Genomes by Support Vector Machines
Source: PLoS Comput Biol. 2009 Apr 3;5(4):e1000338. doi: 10.1371/journal.pcbi.1000338 (PMC2659441; doi:10.1371/journal.pcbi.1000338)
Supplement: Figure S4 — Distribution of sequence identities and size of the sequence sets studied. (A) Training sets. (B) Test sets. (C) Shewanella sequence sets. (0.01 MB PDF) [file pcbi.1000338.s006.pdf]

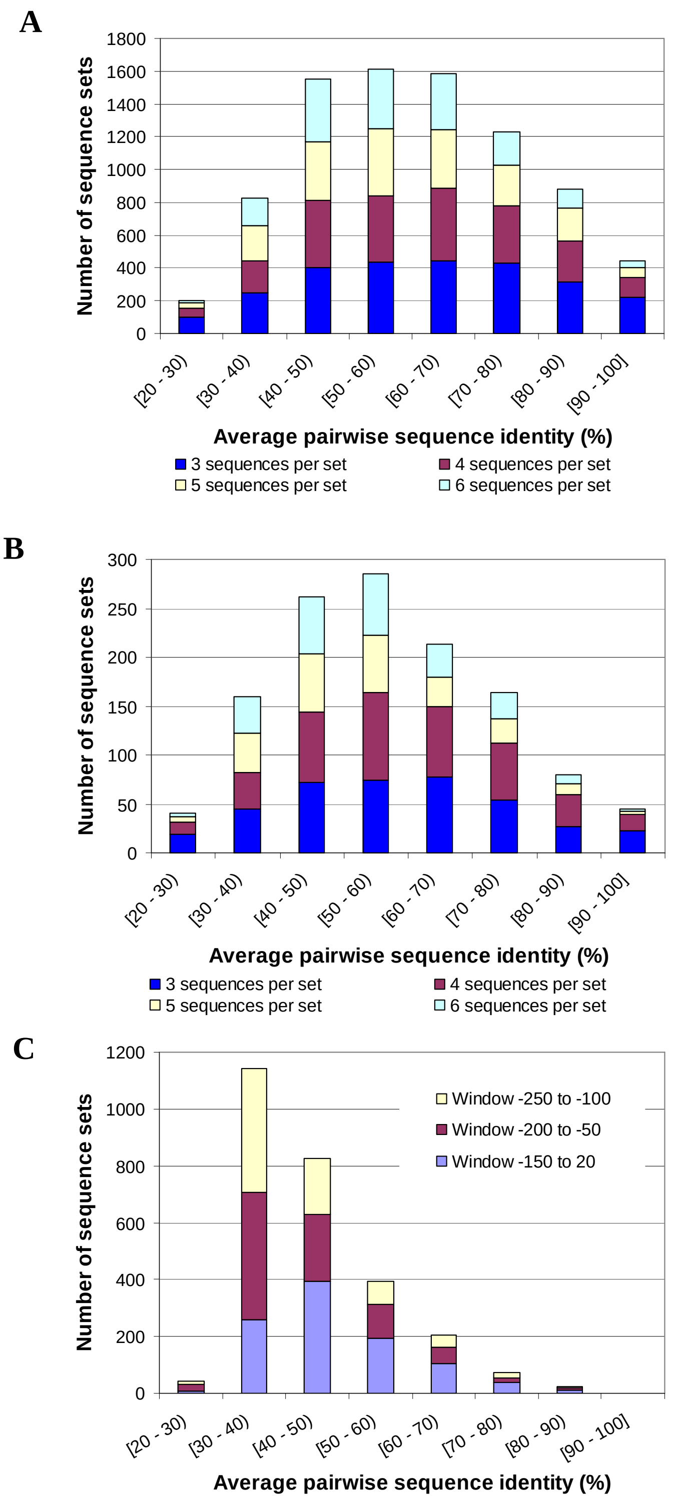

Figure S4. Distribution of sequence identities and size of the sequence sets studied. A. Training sets. B. Test sets. C. *Shewanella* sequence sets.
