# Supplementary material for: Discovering cis-Regulatory RNAs in Shewanella Genomes by Support Vector Machines
Source: PLoS Comput Biol. 2009 Apr 3;5(4):e1000338. doi: 10.1371/journal.pcbi.1000338 (PMC2659441; doi:10.1371/journal.pcbi.1000338)
Supplement: Figure S5 — Flow chart of the genome-wide identification of RNA regulatory motifs/genes using RNA Sampler and RSSVM. (0.01 MB PDF) [file pcbi.1000338.s007.pdf]

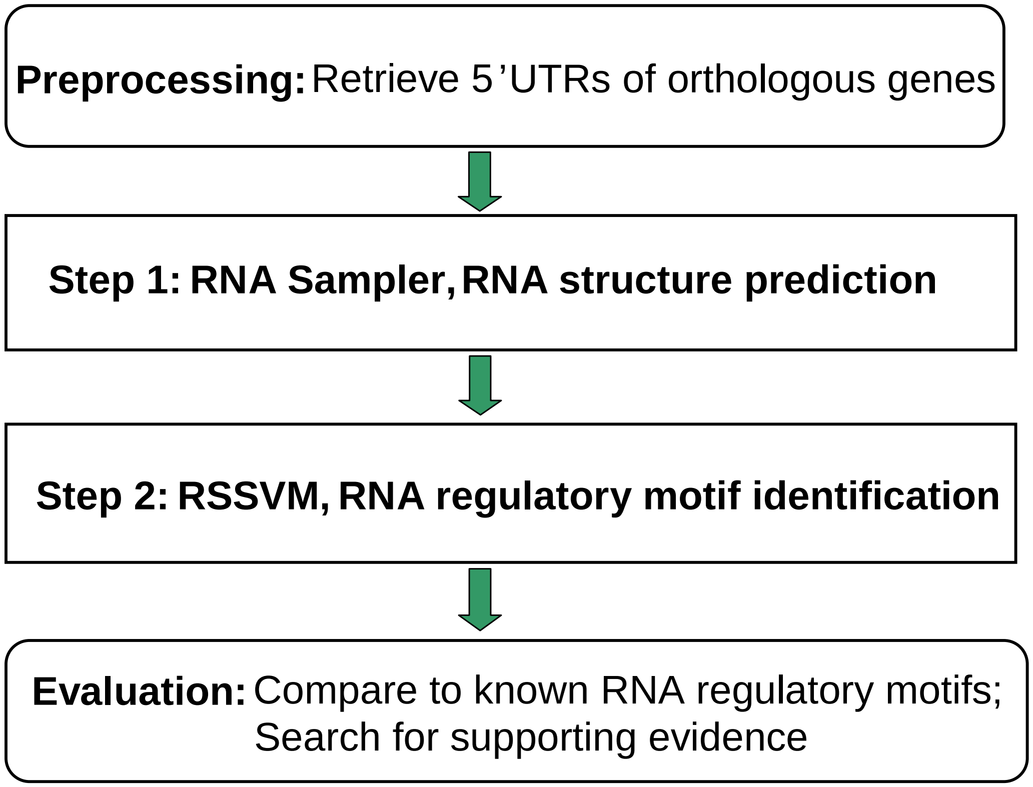

Figure S5. Flow chart of the genome-wide identification of RNA regulatory motifs/genes using RNA Sampler and RSSVM.
