# Supplementary material for: Discovering cis-Regulatory RNAs in Shewanella Genomes by Support Vector Machines
Source: PLoS Comput Biol. 2009 Apr 3;5(4):e1000338. doi: 10.1371/journal.pcbi.1000338 (PMC2659441; doi:10.1371/journal.pcbi.1000338)
Supplement: Table S1 — The prediction sensitivities and false positive rates of RSSVM, RNAz, Dynalign+LIBSVM and QRNA on test sets with different sequence identities. Different P-value cutoffs were used to fairly compare prediction sensitivities of different SVM models at the same FPR level. Numbers in bold fonts are the best results given by all the programs for an identity range. (0.01 MB PDF) [file pcbi.1000338.s008.pdf]

Table S1. The prediction sensitivities and false positive rates of RSSVM, RNAz, Dynalign+LIBSVM and QRNA on test sets with different sequence identities. Different *P*-value cutoffs were used to fairly compare prediction sensitivities of different SVM models at the same FPR level. Numbers in bold fonts are the best results given by all the programs for an identity range.

Identity range (%)	Number of sets	RSSVM vs RNAz and Dynalign+LIBSVM[a]						RSSVM vs RNAz, Dynalign+LIBSVM and QRNA[b]							
		Sensitivity			False Positive Rate			Sensitivity				False Positive Rate			
		RSSVM (*P*>0.95)	RNAz (*P*>0.50)	Dynalign+ LIBSVM (*P*>0.99)	RSSVM (*P*>0.95)	RNAz (*P*>0.50)	Dynalign+ LIBSVM (*P*>0.99)	RSSVM (*P*>0.58)	RNAz (*P*>0.30[c])	Dynalign+ LIBSVM (*P*>0.82)	QRNA	RSSVM (*P*>0.58)	RNAz (*P*>0.30)	Dynalign+ LIBSVM (*P*>0.82)	QRNA
[20, 30)	39	**0.537**	0.220	0.185	**0.000**	**0.000**	0.010	**0.756**	**0.756**	0.316	0.130	0.049	0.220	0.018	**0.017**
[30, 40)	210	**0.734**	0.576	0.268	**0.000**	0.013	0.008	0.861	**0.905**	0.444	0.202	**0.013**	0.209	0.016	0.014
[40, 50)	342	**0.734**	0.624	0.287	**0.004**	0.008	0.018	**0.901**	0.707	0.509	0.349	0.027	0.027	0.034	**0.019**
[50, 60)	361	**0.761**	0.691	0.287	0.007	**0.004**	0.010	**0.905**	0.712	0.513	0.601	0.046	**0.007**	0.025	0.041
[60, 70)	268	0.682	**0.766**	0.438	**0.005**	0.014	0.033	**0.874**	0.804	0.69	0.690	0.037	**0.014**	0.038	0.093
[70, 80)	226	0.579	**0.634**	0.569	**0.006**	0.012	0.011	**0.774**	0.720	0.751	0.751	0.079	**0.024**	0.03	0.097
[80, 90)	143	0.500	**0.662**	0.604	0.037	**0.025**	0.035	**0.838**	0.688	0.777	0.804	0.100	**0.087**	0.104	0.122
[90, 100]	97	0.511	**0.756**	0.607	0.044	**0.000**	0.139	**0.822**	0.756	0.805	0.676	0.244	**0.044**	0.316	0.120
Total	1686	**0.68**	0.65	0.32	0.01	0.01	0.02	**0.86**	0.75	0.51	0.50	0.05	0.05	0.05	0.05

[a] Comparing the sensitivities of RSSVM, RNAz and Dynalign+LIBSVM on sequences sets with different identities at the same overall FPR of 0.01 (0.02 is the lowest possible FPR that can be achieved for Dynalign+LIBSVM;
[b] Comparing the sensitivities of RSSVM, RNAz, Dynalign+LIBSVM and QRNA on sequences sets with different identities at the same overall FPR of 0.05;
[c] In practice, we usually do not use a *P*-value lower than 0.5 as a cutoff.
