# Supplementary material for: Discovering cis-Regulatory RNAs in Shewanella Genomes by Support Vector Machines
Source: PLoS Comput Biol. 2009 Apr 3;5(4):e1000338. doi: 10.1371/journal.pcbi.1000338 (PMC2659441; doi:10.1371/journal.pcbi.1000338)
Supplement: Table S2 — Top 166 predicted regulatory RNAs by RSSVM. (0.06 MB PDF) [file pcbi.1000338.s009.pdf]

Table S2. Top 166 predicted regulatory RNAs by RSSVM.

Rank	GI	RSSVM	RNAz	QRNA	Terminator (T) Attenuator (A)	Gene Name	Gene Product	Knowledge of Regulation	Reference
1	**24349136**	1.000	1.000	Q(FP)[a]	T A	trpE	anthranilate synthase component I	RF00513 Trp_leader	Rfam
2	**24345897**	1.000	1.000	Q	T A	rsd	regulator of sigma D	Leader peptide, and transcription attenuator	(Vitreschak *et al*. 2004)
3	**24346870**	1.000	1.000	Q(FP)	T A	SO1202	conserved hypothetical protein	RF00005 tRNA	Rfam
4	**24351250**	1.000	0.998	Q(FP)	T A	SO4727	conserved hypothetical protein	RF00558 L20_leader	Rfam
5	**24350784**	1.000	0.998	Q	T -	ilvG	acetolactate synthase II, large subunit		
6	**24347627**	1.000	0.998		T A	ppiD	peptidyl-prolyl cis-trans isomerase D	RF00506 Thr_leader	Rfam
7	**24349634**	1.000	0.997	Q	T A	thrA	aspartokinasTomoserine dehydrogenase, threonine-sensitive	RF00506 Thr_leader	Rfam
8	**24347975**	1.000	0.997	Q(FP)	T A	hisG	ATP phosphoribosyltransferase	RF00514 His_leader	Rfam
9	**24349200**	1.000	0.996	Q	T A	SO3069	conserved hypothetical protein		
10	**24349922**	1.000	0.992	Q	T A	SO3649	GTP-binding protein, GTP1/Obg family		
11	**24347698**	1.000	0.988	Q	T A	rmf	ribosome modulation factor		
12	**24351271**	1.000	0.916		T A	SO4742	transcriptional regulator, DeoR family		
13	**24346855**	1.000	0.643		T A	greA	transcription elongation factor GreA		
14	**24348820**	1.000	0.634		T A	SO2744	helicase		
15	**24348163**	1.000	0.542		T A	SO2223	peptidase, putative		
16	**24347418**	1.000	0.523			rpsB	ribosomal protein S2	RF00127 t44 RNA	Rfam
17	**24346570**	1.000	0.507		T A	ldhA	D-lactate dehydrogenase	Possible post-transcriptional effect	(Jiang *et al*. 2001)
18	**24346427**	1.000	0.409		T A	SO0858	sodium:alanine symporter family protein		

Table S2. Top 166 predicted regulatory RNAs by RSSVM (continued 1).

Rank	GI	RSSVM	RNAz	QRNA	Terminator (T) Attenuator (A)	Gene Name	Gene Product	Knowledge of Regulation	Reference
19	**24346216**	1.000	0.319		T A	SO0695	glutathione-regulated potassium-efflux system protein KefC, putative		
20	**24348228**	1.000	0.272		T A	ndk	nucleoside diphosphate kinase		
21	**24349359**	1.000	0.223			SO3191	chain length determinant protein		
22	**24348648**	1.000	0.150			SO2602	conserved hypothetical protein		
23	**24348431**	1.000	0.105		T -	aspS	aspartyl-tRNA synthetase	tRNA synthetase leader	
24	**24350063**	1.000	0.996	Q		SO3769	conserved hypothetical protein		
25	**24348233**	1.000	0.904			ilvI	acetolactate synthase III, large subunit	Leader peptide, and transcription attenuator	(Vitreschak *et al*. 2004)
26	**24349427**	1.000	0.862			flgB	flagellar basal-body rod protein FlgB	Putative GEMM element	(Weinberg *et al*. 2007)
27	**24348700**	1.000	0.241			aroH	phospho-2-dehydro-3-deoxyheptonate aldolase, trp-sensitive	Possible transcription termination	(Zurawski *et al*. 1981)
28	**24346058**	1.000	0.037		T A	plsC	1-acyl-sn-glycerol-3-phosphate acyltransferase	Leader peptide, and transcription attenuator	(Vitreschak *et al*. 2004)
29	**24345795**	1.000	0.850	Q	T -	SO0354	sodium/calcium exchanger		
30	**24345909**	1.000	0.999			purH	phosphoribosylaminoimidazolecarboxamide formyltransferase/IMP cyclohydrolase		
31	**24347794**	1.000	1.000	Q	T A	sucA	2-oxoglutarate dehydrogenase, E1 component		
32	**24349814**	1.000	0.570		T -	gshA	glutamate--cysteine ligase		
33	**24349842**	1.000	0.974	Q		SO3582	methyl-accepting chemotaxis protein		
34	**24346699**	1.000	1.000	Q		SO1071	conserved hypothetical protein	RF00080 yybP-ykoY	Rfam
35	**24350656**	1.000	0.998	Q	T A	leuA	2-isopropylmalate synthase	Leader peptide, and transcription attenuator	(Vitreschak *et al*. 2004)

Table S2. Top 166 predicted regulatory RNAs by RSSVM (continued 2).

Rank	GI	RSSVM	RNAz	QRNA	Terminator (T) Attenuator (A)	Gene Name	Gene Product	Knowledge of Regulation	Reference
36	**24348336**	1.000	0.235			ccoN	cytochrome c oxidase, cbb3-type, subunit I		
37	**24347061**	1.000	0.990	Q	T A	lepB	signal peptidase I		
38	**24349573**	1.000	0.455			SO3364	conserved hypothetical protein		
39	**24347085**	1.000	0.997	Q	T A	pheA	chorismate mutase/prephenate dehydratase	RF00513 Trp_leader	Rfam
40	**24349420**	1.000	0.868	Q		flgF	flagellar basal-body rod protein FlgF		
41	**24345882**	1.000	0.094			pdhR	pyruvate dehydrogenase complex repressor	PdhR-box in *E. coli*	(Ogasawara *et al*. 2007)
42	**24350809**	1.000	0.454			SO4366	conserved hypothetical protein		
43	**24349259**	1.000	0.914	Q	T A	tgt	queuine tRNA-ribosyltransferase		
44	**24348337**	1.000	0.939			SO2365	conserved hypothetical protein		
45	**24349640**	1.000	0.750		T A	SO3418	hypothetical protein		
46	**24347081**	1.000	0.095		T A	hcp	prismane protein		
47	**24350912**	1.000	0.995			moaA	molybdenum cofactor biosynthesis protein A		
48	**24345525**	1.000	0.407			ribB	3,4-dihydroxy-2-butanone 4-phosphate synthase		
49	**24345845**	1.000	0.864			SO0394	conserved hypothetical protein		
50	**24348221**	1.000	0.940		T -	rimK-2	ribosomal protein S6 modification protein		
51	**24345862**	1.000	0.997	Q		rho	transcription termination factor Rho		
52	**24348056**	1.000	0.893		T -	adhE	aldehyde-alcohol dehydrogenase	Stem-loop for occupying RBS in *E. coli*	(Membrillo-Hernandez and Lin 1999)
53	**24351269**	1.000	0.018		T A	SO4740	conserved hypothetical protein		
54	**24348439**	1.000	0.390		- A	SO2438	transcriptional regulator, LysR family		

Table S2. Top 166 predicted regulatory RNAs by RSSVM (continued 3).

Rank	GI	RSSVM	RNAz	QRNA	Terminator (T) Attenuator (A)	Gene Name	Gene Product	Knowledge of Regulation	Reference
55	**24346560**	1.000	0.196			ahpC	alkyl hydroperoxide reductase, C subunit		
56	**24349996**	1.000	0.459		T -	SO3711	conserved hypothetical protein		
57	**24345468**	1.000	0.476			hutI	imidazolonepropionase		
58	**24349731**	1.000	0.571			mexE	RND multidrug efflux membrane fusion protein MexE		
59	**24349583**	1.000	0.361			SO3371	conserved hypothetical protein		
60	**24349939**	1.000	0.397			fadD-2	long-chain-fatty-acid--CoA ligase		
61	**24347619**	1.000	0.947		T -	tig	trigger factor		
62	**24345537**	1.000	0.465			SO0150	lipoprotein, putative		
63	**24347612**	1.000	0.012	Q	T A	glnS	glutaminyl-tRNA synthetase	tRNA synthetase leader	(Cheung *et al*. 1985)
64	**24346616**	1.000	1.000	Q		SO1007	conserved hypothetical protein	RF00168 Lysine	Rfam
65	**24347626**	0.999	1.000	Q		SO1797	DNA-binding protein, HU family		
66	**24347658**	0.999	0.988	Q		SO1824	conserved hypothetical protein		
67	**24349607**	0.999	0.997	Q		SO3392	oxidoreductase, FMN-binding		
68	**24348243**	0.999	0.424			SO2286	sulfate permease family protein		
69	**24350411**	0.999	0.435			SO4048	cytochrome c family protein		
70	**24349974**	0.999	0.530			SO3694	conserved hypothetical protein		
71	**24350483**	0.999	0.918		T A	mshC	MSHA pilin protein MshC		
72	**24350423**	0.999	0.997			metJ	met repressor		
73	**24348868**	0.999	0.240	Q	T A	rne	ribonuclease E	RF00370 sroD RNA	Rfam
74	**24348465**	0.999	0.641	Q	T A	SO2458	transporter, putative		
75	**24347241**	0.999	0.419			SO1489	hypothetical protein		
76	**24346750**	0.999	0.000		- A	bfr2	bacterioferritin subunit 2		
77	**24345794**	0.999	0.535		T -	SO0353	TPR domain protein		
78	**24345538**	0.999	0.526		T A	SO0151	conserved hypothetical protein		
79	**24346342**	0.999	0.417			SO0793	hypothetical protein		
80	**24347038**	0.999	0.810	Q		SO1331	MutT/nudix family protein		
81	**24348153**	0.999	0.669			SO2216	sensory box protein		

Table S2. Top 166 predicted regulatory RNAs by RSSVM (continued 4).

Rank	GI	RSSVM	RNAz	QRNA	Terminator (T) Attenuator (A)	Gene Name	Gene Product	Knowledge of Regulation	Reference
82	**24348420**	0.999	0.094			SO2427	TonB-dependent receptor, putative		
83	**24347590**	0.998	0.451		T A	SO1769	glutamate decarboxylase, putative	Possible post-transcriptional regulation in *S. oneidensis*	(Yang *et al*. 2008)
84	**24348152**	0.998	0.474		T A	SO2215	sun protein, putative		
85	**24347329**	0.998	0.288	Q	T A	SO1560	phosphate-binding protein		
86	**24349540**	0.998	0.435		T A	SO3337	conserved domain protein		
87	**24349811**	0.998	0.697		T -	SO3556	cyclic nucleotide phosphodiesterase, putative		
88	**24345631**	0.998	0.999	Q(FP)	T -	rpoB	DNA-directed RNA polymerase, beta subunit	Transcriptional attenuation	(Ishihama and Fukuda 1980; Steward and Linn 1992)
89	**24350422**	0.998	0.100	Q		metB	cystathionine gamma-synthase		
90	**24349961**	0.998	0.424	Q		SO3683	coniferyl aldehyde dehydrogenase	Ribosomal protein leader	Rfam
91	**24345625**	0.998	1.000			rplJ	ribosomal protein L10		
92	**24350610**	0.997	0.259			ubiE	ubiquinone/menaquinone biosynthesis methlytransferase UbiE		
93	**24346037**	0.997	0.678			SO0547	conserved hypothetical protein	RF00522 PreQ1	Rfam
94	**24350762**	0.997	0.317			SO4329	conserved hypothetical protein		
95	**24347883**	0.996	0.396			ushA	5'-nucleotidase		
96	**24349570**	0.996	0.418			SO3361	conserved hypothetical protein		
97	**24348938**	0.996	0.545			SO2846	conserved hypothetical protein		
98	**24349291**	0.996	0.345			SO3140	thymidine kinase		
99	**24347300**	0.995	0.103			SO1538	isocitrate dehydrogenase, NAD-dependent		
100	**24348781**	0.995	0.986		- A	SO2715	TonB-dependent receptor	RF00059 TPP	Rfam

Table S2. Top 166 predicted regulatory RNAs by RSSVM (continued 5).

Rank	GI	RSSVM	RNAz	QRNA	Terminator (T) Attenuator (A)	Gene Name	Gene Product	Knowledge of Regulation	Reference
101	**24348214**	0.994	0.964			SO2263	Rrf2 family protein		
102	**24350917**	0.994	0.439			SO4454	methyl-accepting chemotaxis protein		
103	**24349041**	0.994	0.982	Q		SO2935	oxidoreductase, short-chain dehydrogenase/reductase family		
104	**24346274**	0.994	0.256		T A	SO0740	melanin biosynthesis protein TyrA, putative		
105	**24349015**	0.994	0.274	Q	T -	pflB	formate acetyltransferase	Possible post-transcriptional regulation	(Beliaev *et al*. 2002)
106	**24350214**	0.994	0.688			SO3896	outer membrane porin, putative	Post-transcriptional regulation in *S. oneidensis*	(Maier and Myers 2004)
107	**24351072**	0.993	0.890	Q		rpoH	RNA polymerase sigma-32 factor		
108	**24347836**	0.993	0.064			SO1963	conserved hypothetical protein		
109	**24345633**	0.992	0.988	Q	T A	rpsL	ribosomal protein S12	Ribosomal protein leader	(Yao *et al*. 2007)
110	**24349105**	0.992	0.359			SO2990	prophage LambdaSo, transcriptional regulator, Cro/CI family		
111	**24346963**	0.992	0.599			SO1274	conserved hypothetical protein		
112	**24349403**	0.990	0.179		T -	fliE	flagellar hook-basal body complex protein FliE	Putative GEMM element	(Weinberg *et al*. 2007)
113	**24345796**	0.990	0.385			SO0355	AMP-binding protein		
114	**24347791**	0.990	0.618			sdhC	succinate dehydrogenase, cytochrome b556 subunit		
115	**24346525**	0.989	0.015			fba	fructose-bisphosphate aldolase, class II, Calvin cycle subtype		
116	**24345418**	0.989	0.545			secB	protein-export protein SecB		
117	**24350326**	0.989	0.765	Q(FP)		lysC	aspartokinase III, lysine-sensitive	RF00168 Lysine	Rfam

Table S2. Top 166 predicted regulatory RNAs by RSSVM (continued 6).

Rank	GI	RSSVM	RNAz	QRNA	Terminator (T) Attenuator (A)	Gene Name	Gene Product	Knowledge of Regulation	Reference
118	**24349226**	0.989	0.057	Q(FP)		SO3089	fatty oxidation complex, beta subunit		
119	**24346986**	0.989	0.320			folB	dihydroneopterin aldolase		
120	**24348446**	0.989	0.420			thiC	thiamin biosynthesis protein ThiC	RF00059 TPP	Rfam
121	**24348979**	0.988	0.704	Q	- A	SO2882	conserved hypothetical protein		
122	**24347613**	0.988	0.267			SO1787	conserved hypothetical protein		
123	**24350786**	0.988	0.277	Q		ilvC	ketol-acid reductoisomerase		
124	**24345621**	0.987	0.108		T -	secE	preprotein translocase, SecE subunit	RNaseIII sites in the leader sequence of SecE in *E. coli*	(Chow and Dennis 1994)
125	**24347051**	0.987	0.969			nadB	L-aspartate oxidase	RF00522 PreQ1	Rfam
126	**24346706**	0.987	0.353	Q	T A	SO1075	conserved hypothetical protein		
127	**24349968**	0.987	0.493			SO3687	curli production assembly/transport component CsgE, putative		
128	**24349655**	0.986	0.932	Q		recA	recA protein		
129	**24348006**	0.985	0.006			hoxK	quinone-reactive Ni/Fe hydrogenase, small subunit precursor		
130	**24348892**	0.985	0.366			SO2805	conserved hypothetical protein		
131	**24345589**	0.982	0.512		T A	SO0190	MutT/nudix family protein		
132	**24351101**	0.982	0.994		T -	SO4606	cytochrome c oxidase, subunit II		
133	**24346318**	0.982	0.690			SO0774	5-formyltetrahydrofolate cyclo-ligase family protein	RF00013 6S RNA	Rfam
134	**24348931**	0.981	0.432			SO2842	peptidase, M23/M37 family		
135	**24350327**	0.980	0.029	Q	- A	SO3987	conserved hypothetical protein		
136	**24349297**	0.980	0.850		- A	etfB	electron transfer flavoprotein, beta subunit		
137	**24346527**	0.980	0.378			nhaD	Na+/H+ antiporter		

Table S2. Top 166 predicted regulatory RNAs by RSSVM (continued 7).

Rank	GI	RSSVM	RNAz	QRNA	Terminator (T) Attenuator (A)	Gene Name	Gene Product	Knowledge of Regulation	Reference
138	**24348231**	0.979	0.402			ibpA	16 kDa heat shock protein A		
139	**24347681**	0.978	0.423			SO1844	extracellular nuclease, putative		
140	**24349463**	0.978	0.966	Q		SO3278	conserved hypothetical protein		
141	**24348630**	0.977	0.006	Q		SO2591	conserved hypothetical protein		
142	**24350000**	0.977	0.410			SO3715	oxygen-insensitive NAD(P)H nitroreductase		
143	**24350064**	0.976	0.858	Q	T A	SO3770	conserved hypothetical protein TIGR00153		
144	**24351130**	0.975	0.277		- A	SO4628	sulfatase		
145	**24346130**	0.975	0.040			crp	catabolite gene activator		
146	**24346384**	0.972	0.342			SO0826	conserved hypothetical protein		
147	**24347716**	0.972	0.456		T -	speA	biosynthetic arginine decarboxylase	Possible post-transcriptional regulation in *S. oneidensis*	(Yang *et al*. 2008)
148	**24348388**	0.971	0.017			cmk	cytidylate kinase		
149	**24347035**	0.971	0.393			SO1328	transcriptional regulator, LysR family		
150	**24350162**	0.971	0.796		T A	sfcA	malate oxidoreductase		
151	**24349576**	0.971	0.367			SO3367	conserved hypothetical protein TIGR00091		
152	**24345923**	0.970	0.418			fkbP-1	peptidyl-prolyl cis-trans isomerase FkbP		
153	**24351092**	0.967	0.333		T A	SO4600	antigen, putative		
154	**24345880**	0.967	0.415		T A	ampD	AmpD protein		
155	**24349361**	0.965	0.450			SO3193	polysaccharide biosynthesis protein		
156	**24350020**	0.964	0.413		- A	SO3733	hypothetical protein		
157	**24346579**	0.964	0.136			SO0975	hypothetical protein		
158	**24348690**	0.961	0.426			SO2639	hypothetical protein		

Table S2. Top 166 predicted regulatory RNAs by RSSVM (continued 8).

Rank	GI	RSSVM	RNAz	QRNA	Terminator (T) Attenuator (A)	Gene Name	Gene Product	Knowledge of Regulation	Reference
159	**24345372**	0.960	0.440		T A	SO0019	conserved hypothetical protein		
160	**24350679**	0.959	0.376			folE	GTP cyclohydrolase I		
161	**24347079**	0.958	0.524			aroF	phospho-2-dehydro-3-deoxyheptonate aldolase, tyr-sensitive		
162	**24348818**	0.957	0.191			SO2742	sensor histidine kinase/response regulator		
163	**24349925**	0.956	0.102			rplU	ribosomal protein L21		
164	**24348689**	0.954	0.897		T A	ldh	leucine dehydrogenase		
165	**24346880**	0.954	0.623		- A	SO1210	TPR domain protein		
166	**24349795**	0.953	0.409		- A	SO3545	OmpA family protein		

[a]. “Q” represents QRNA predictions that fit the “RNA” model in at least two pairwise alignments. “FP” indicates that the shuffled sequences were also identified as “RNA” by QRNA.

References

Beliaev AS, Thompson DK, Khare T, Lim H, Brandt CC et al. (2002) Gene and protein expression profiles of Shewanella oneidensis during anaerobic growth with different electron acceptors. Omics 6(1): 39-60.

Cheung AY, Watson L, Soll D (1985) Two control systems modulate the level of glutaminyl-tRNA synthetase in Escherichia coli. Journal of bacteriology 161(1): 212-218.

Chow J, Dennis PP (1994) Coupling between mRNA synthesis and mRNA stability in Escherichia coli. Molecular microbiology 11(5): 919-931.

Ishihama A, Fukuda R (1980) Autogenous and post-transcriptional regulation of RNA polymerase synthesis. Molecular and cellular biochemistry 31(3): 177-196.

Jiang GR, Nikolova S, Clark DP (2001) Regulation of the ldhA gene, encoding the fermentative lactate dehydrogenase of Escherichia coli. Microbiology (Reading, England) 147(Pt 9): 2437-2446.

Maier TM, Myers CR (2004) The outer membrane protein Omp35 affects the reduction of Fe(III), nitrate, and fumarate by Shewanella oneidensis MR-1. BMC microbiology 4: 23.

Membrillo-Hernandez J, Lin EC (1999) Regulation of expression of the adhE gene, encoding ethanol oxidoreductase in Escherichia coli: transcription from a downstream promoter and regulation by fnr and RpoS. Journal of bacteriology 181(24): 7571-7579.

Ogasawara H, Ishida Y, Yamada K, Yamamoto K, Ishihama A (2007) PdhR (pyruvate dehydrogenase complex regulator) controls the respiratory electron transport system in Escherichia coli. Journal of bacteriology 189(15): 5534-5541.

Steward KL, Linn T (1992) Transcription frequency modulates the efficiency of an attenuator preceding the rpoBC RNA polymerase genes of Escherichia coli: possible autogenous control. Nucleic acids research 20(18): 4773-4779.

Vitreschak AG, Lyubetskaya EV, Shirshin MA, Gelfand MS, Lyubetsky VA (2004) Attenuation regulation of amino acid biosynthetic operons in proteobacteria: comparative genomics analysis. FEMS microbiology letters 234(2): 357-370.

Weinberg Z, Barrick JE, Yao Z, Roth A, Kim JN et al. (2007) Identification of 22 candidate structured RNAs in bacteria using the CMfinder comparative genomics pipeline. Nucleic Acids Res 35(14): 4809-4819.

Yang Y, Harris DP, Luo F, Wu L, Parsons AB et al. (2008) Characterization of the Shewanella oneidensis Fur gene: roles in iron and acid tolerance response. BMC genomics 9 Suppl 1: S11.

Yao Z, Barrick J, Weinberg Z, Neph S, Breaker R et al. (2007) A computational pipeline for high-throughput discovery of cis-regulatory noncoding RNA in prokaryotes. PLoS computational biology 3(7): e126.

Zurawski G, Gunsalus RP, Brown KD, Yanofsky C (1981) Structure and regulation of aroH, the structural gene for the tryptophan-repressible 3-deoxy-D-arabino-heptulosonic acid-7-phosphate synthetase of Escherichia coli. Journal of molecular biology 145(1): 47-73.
