# Supplementary material for: Discovering cis-Regulatory RNAs in Shewanella Genomes by Support Vector Machines
Source: PLoS Comput Biol. 2009 Apr 3;5(4):e1000338. doi: 10.1371/journal.pcbi.1000338 (PMC2659441; doi:10.1371/journal.pcbi.1000338)
Supplement: Table S3 — Comparisons between different RNA motif identification algorithms. (0.01 MB PDF) [file pcbi.1000338.s010.pdf]

Table S3. Comparisons between different RNA motif identification algorithms.

	**RSSVM**	**RNAz**	**Dynalign+LIBSVM**	**QRNA**
Structure prediction	RNA Sampler	RNAalifold	Dynalign	N/A
Algorithm	SVM	SVM	SVM	Probabilistic Model
Input	Unaligned sequences	Sequence alignments	Two unaligned sequences	Pairwise sequence alignments
Feature vector	*Z, SCI* [a], *I, MI, id, n*	*Z, SCI* [a], *id, n*	$\Delta G_{total}, L, f^{A}_{seq1}, f^{U}_{seq1}, f^{C}_{seq1}, f^{A}_{seq2}, f^{U}_{seq2}, f^{C}_{seq2},$	N/A
Training sets	All bacterial RNA motif families from Rfam<br>Sequence identity: 20% -90%	Rfam families from Rfam<br>Sequence identities: > 50%	tRNAs and rRNAs	tRNAs and rRNAs
Output	Common RNA secondary structures, classification *P*-value	Common RNA secondary structures, classification *P*-value	Common RNA secondary structures, classification *P*-value	Classification of RNA, Coding or Null

[a]. The *SCI* score in RNAz is calculated from the common structure predicted by RNAalifold. It also considers covariance between base pairs. The *SCI* score in RSSVM is calculated from the common structure predicted by RNA Sampler.
